# The missing pieces for better future predictions in subarctic ecosystems: A Torneträsk case study

**DOI:** 10.1007/s13280-020-01381-1

**Published:** 2020-09-13

**Authors:** Didac Pascual, Jonas Åkerman, Marina Becher, Terry V. Callaghan, Torben R. Christensen, Ellen Dorrepaal, Urban Emanuelsson, Reiner Giesler, Dan Hammarlund, Edward Hanna, Annika Hofgaard, Hongxiao Jin, Cecilia Johansson, Christer Jonasson, Jonatan Klaminder, Jan Karlsson, Erik Lundin, Anders Michelsen, David Olefeldt, Andreas Persson, Gareth K. Phoenix, Zofia Rączkowska, Riikka Rinnan, Lena Ström, Jing Tang, Ruth K. Varner, Philip Wookey, Margareta Johansson

**Affiliations:** 1 Department of Physical Geography and Ecosystem Science, Lund University, Sölvegatan 12, 223 62 Lund, Sweden; 2 Geological Survey of Sweden, Box 670, 751 28 Uppsala, Sweden; 3 Alfred Denny Building, University of Sheffield, Western Bank, Sheffield, S10 2TN UK; 4 Department of Botany, National Research Tomsk State University, 36 Lenin Ave., Tomsk, Russia 634050; 5 Department of Bioscience, Faculty of Technical Sciences, Aarhus University, Frederiksborgvej 399, 4000 Roskilde, Denmark; 6 Swedish Biodiversity Centre, Swedish University of Agricultural Sciences, Mobergavägen 19, 373 54 Senoren, Sweden; 7 Climate Impacts Research Centre, Department of Ecology and Environmental Science, Umeå University, 90187 Umeå, Sweden; 8 Department of Geology, Lund University, Sölvegatan 12, 223 62 Lund, Sweden; 9 School of Geography, Think Tank, Ruston Way, Lincoln, LN6 7FL UK; 10 Norwegian Institute for Nature Research, Torgarden, P.O. Box 5685, 7485 Trondheim, Norway; 11 Department of Environmental Engineering, Technical University of Denmark, 2800 Kgs., Lyngby, Denmark; 12 Department of Earth Sciences, Uppsala University, Villavägen 16, 752 36 Uppsala, Sweden; 13 Department of Social and Economic Geography, Uppsala University, Box 513, 751 20 Uppsala, Sweden; 14 Swedish Polar Research Secretariat, Luleå tekniska universitet, 971 87 Luleå, Sweden; 15 Department of Biology, University of Copenhagen, Universitetsparken 15, 2100 Copenhagen Ø, Denmark; 16 Center for Permafrost (CENPERM), University of Copenhagen, Øster Voldgade 10, 1350 Copenhagen K, Denmark; 17 Department of Renewable Resources, University of Alberta, 751 General Services Building, Edmonton, T6G 2H1 Canada; 18 Department of Animal and Plant Sciences, University of Sheffield, Western Bank, Sheffield, S10 2TN UK; 19 Department of Geoenvironmental Research, Institute of Geography and Spatial Organisation PAS, Św. Jana 22, 31-018 Kraków, Poland; 20 Department of Earth Sciences, University of New Hampshire, Morse Hall Rm 455, 8 College Rd., Durham, NH 03824 USA; 21 Biology and Environmental Sciences, School of Natural Sciences, University of Stirling, Stirling, FK9 4LA Scotland UK

**Keywords:** Abiotic drivers, Arctic and subarctic, Biotic drivers, Ecosystem change, Research priorities

## Abstract

Arctic and subarctic ecosystems are experiencing substantial changes in hydrology, vegetation, permafrost conditions, and carbon cycling, in response to climatic change and other anthropogenic drivers, and these changes are likely to continue over this century. The total magnitude of these changes results from multiple interactions among these drivers. Field measurements can address the overall responses to different changing drivers, but are less capable of quantifying the interactions among them. Currently, a comprehensive assessment of the drivers of ecosystem changes, and the magnitude of their direct and indirect impacts on subarctic ecosystems, is missing. The Torneträsk area, in the Swedish subarctic, has an unrivalled history of environmental observation over 100 years, and is one of the most studied sites in the Arctic. In this study, we summarize and rank the drivers of ecosystem change in the Torneträsk area, and propose research priorities identified, by expert assessment, to improve predictions of ecosystem changes. The research priorities identified include understanding impacts on ecosystems brought on by altered frequency and intensity of winter warming events, evapotranspiration rates, rainfall, duration of snow cover and lake-ice, changed soil moisture, and droughts. This case study can help us understand the ongoing ecosystem changes occurring in the Torneträsk area, and contribute to improve predictions of future ecosystem changes at a larger scale. This understanding will provide the basis for the future mitigation and adaptation plans needed in a changing climate.

**Electronic supplementary material:**

The online version of this article (10.1007/s13280-020-01381-1) contains supplementary material, which is available to authorized users.

## Introduction

Increasing greenhouse gas concentrations in the atmosphere have resulted in a general increase in Earth’s surface temperature during the last decades (IPCC 2013). However, climate change has many facets, including changes in precipitation, snow regime, extreme weather, and biotic events, and these changes occur alongside other anthropogenic drivers, such as changes in land use and pollution. All these drivers interact and therefore it is very complex to predict the future of arctic ecosystems.

In the Arctic, the temperature increase is twice as fast as the global average (Cohen et al. 2014), mostly due to the reduced surface albedo, linked to the declining Arctic sea ice extent (Walsh 2014) and snow cover duration (Brown et al. 2017). This trend is likely to continue throughout the twenty-first century (Collins et al. 2013). Apart from the observed increase in air temperature, a general (although uneven) increase in precipitation, both in the form of rain (IPCC 2013), and in some areas snow (Park et al. 2012), has been observed in the Arctic region over recent decades, a trend that is also projected to continue throughout the twenty-first century (IPCC 2013). Given that arctic and subarctic ecosystems are strongly dependent on, and adapted to, specific climatic conditions, these ongoing and predicted climatic changes could impact their biotic (e.g. vegetation and the carbon cycle) and abiotic (e.g. permafrost, hydrology, and local climate) components.

In addition to the observed long-term changes in temperature and precipitation, the frequency and intensity of extreme events, such as fires, winter warming events, extreme rainfall, severe droughts and insect outbreaks, has also increased in the Arctic during recent decades (e.g. Soja et al. 2007; Kivinen et al. 2017). These short-lasting stochastic events have already caused abrupt impacts on arctic ecosystems (e.g. Phoenix and Bjerke 2016; Sokolov et al. 2016), which could grow under the predicted scenarios of more intense and frequent extreme events (e.g. Vikhamar-Schuler et al. 2016; Young et al. 2017).

However, climate change is not the only driver of ecosystem change in the arctic and subarctic areas (ACIA 2005). Rather, the observed changes result from the combined effect of climate change and other anthropogenic factors that are, in turn, highly dependent on governmental policies, such as reindeer herding, land use changes, and pollution. The total magnitude of the ecosystem changes results from the interactions between the different drivers. These changes could potentially have important implications for ecosystem services of vital importance for the local residents (provisioning services, such as food, freshwater or biomass) and for the global population (regulatory services, such as global carbon and energy budgets). Thus, a better understanding of potential future ecosystem changes is paramount for defining climate change mitigation goals and adaptation strategies.

In order to make predictions of the future dynamics of ecosystems, data gathered through monitoring of specific parameters, and the process understanding gained through manipulation experiments, are combined in ecosystem models (e.g. LPJ-GUESS, Smith et al. 2014). These predictions have been improved over the last decades as more data have become available and more advanced ecosystem models have been developed (e.g. Tang et al. 2015.). Nevertheless, these predictions still hold large uncertainties at all spatial and temporal scales, arising mostly from insufficient data, lack of process understanding, and/or model limitations in representing these interacting and other processes. For example, modelled predictions of tree-line movement on subarctic plains have been over-estimated by up to > 1000 times (e.g. Van Bogaert et al. 2011).

Field measurements mostly address overall responses to some changing drivers, rather than the effect of the different interactions between them. Currently, a comprehensive assessment of the drivers (including their direct and indirect effects) of different changes and the magnitude of their impact on subarctic ecosystems is missing.

The Torneträsk area, in the Swedish subarctic, has an unrivalled history of environmental observation spanning over a century (Callaghan et al. 2010; Jonasson et al. 2012), and syntheses of ecosystem changes (e.g. Callaghan et al. 2013). Studies from the Torneträsk area feature in some 12% of all published papers and 19% of all study citations across the Arctic (Metcalfe et al. 2018), excluding internal Russian studies. In the present study, we aim, based on expert opinion, to (i) summarize and rank, in perceived importance, the drivers (including their direct and indirect impacts) of ecosystem change in the Torneträsk area, and to (ii) propose research priorities that are needed to improve future predictions of ecosystem change in the study area and potentially in other arctic ecosystems. The relatively small size of the Torneträsk area, its great biological and geomorphological diversity, and its unique datasets, present a well-curated microcosm of the Subarctic. Its rapidly-transforming ecosystems can underpin an improved understanding of the ongoing processes and future ecosystem changes at a larger circumpolar scale. This understanding, in turn, will provide the basis for future mitigation and adaptation plans needed in a changing climate.

## Methodology

### Study area

**Fig. 1:**
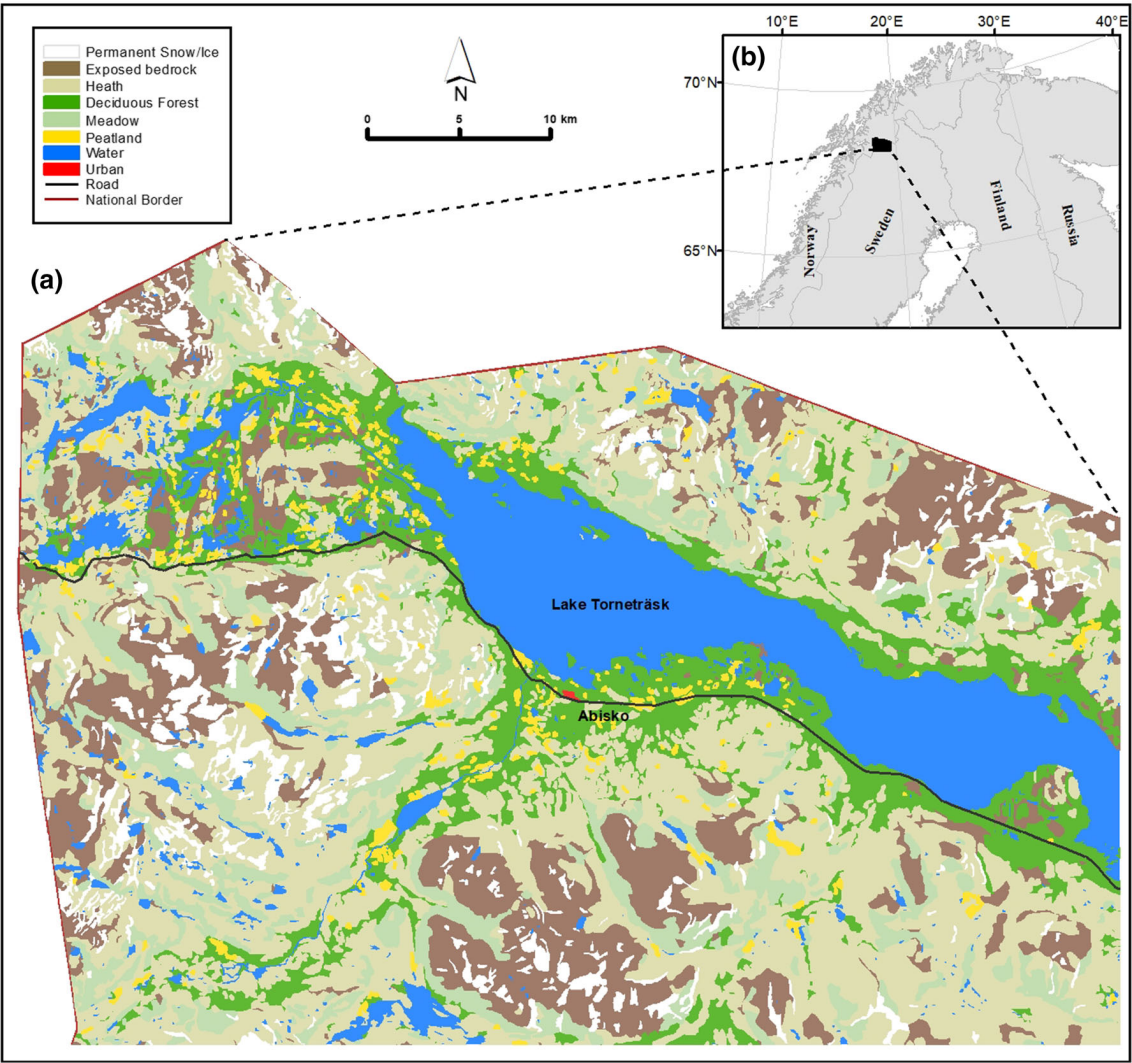
**a** The study area in northernmost Sweden, including dominant land cover classes derived from Lantmäteriet (2006), Sweden. **b** Geographical overview of the study area. Source: Esri; Michael Bauer Research GmbH

The study area includes the northwest part of the Lake Torneträsk catchment, and was delineated to include the climatic, altitudinal, and vegetation gradients occurring in the area (Fig. 1). The region contains highly varied topography, with altitudes ranging between 342 and > 1900 m a.s.l. (Andersson et al. 1996). The climate presents a strong northwest-southeast oceanic-continental gradient, resulting in significant eastward declines in precipitation and winter temperature, caused by increasing distance from the Atlantic Ocean and the strong rain shadow effect caused by the Scandes Mountains. At the Abisko Scientific Research Station (ANS; 385 m a.s.l.), mean annual air temperature (MAAT) increased by 2.5 °C over the period 1913–2006 (Callaghan et al. 2010), and is currently 0.4 °C (ANS 2020). Meteorological data from Abisko Observatory, annual mean 2010–01-01–2019–12-31). Total annual precipitation ranges from > 1000 mm in the north-western areas to ~ 300 mm in the central and southeastern parts of the study area. At the ANS, the mean annual precipitation for the period 2010–2019 was 357 mm, 19% higher than the 301 mm corresponding to the period 1961–1990 (ANS 2020). Meteorological data from Abisko Observatory, annual mean 2010–01-01–2019–12-31).

Vegetation in the area varies with altitude, and is also dependent on hydrology. In the lowlands, birch (*Betula pubescens* var *pumila* L.)-dominated deciduous forests alternate with wetland areas composed of shrubs (e.g. *Vaccinium uliginosum* L.), mosses (e.g. *Sphagnum fuscum* (Schimp.)), lichens (e.g. *Cetraria cucullata*) and graminoids (e.g. *Eriophorum vaginatum* L.) (Johansson et al. 2013), which are expanding in areas of permafrost degradation (e.g. Christensen et al. 2004). Birch-dominated forests occur below an approximate altitudinal limit of 600 and 800 m a.s.l. in the western and eastern parts of the Torneträsk area, respectively (Wielgolaski et al. 2005), and have expanded their altitudinal and latitudinal ranges during recent decades (Callaghan et al. 2013. and references therein). Above the tree-line, the vegetation is mostly composed of dwarf shrub heathland (e.g. *Empetrum hermaphroditum*, and *Vaccinium* species), meadows dominated by sedges, herbs, and graminoids (Sundqvist et al. 2013), and snowbed communities (Björk et al. 2007), which, except for the latter, have increased in areal extent and species richness over the recent decades (e.g. Hedenås et al. 2012). Vegetation cover tends to disappear as elevation increases and where bedrock is exposed or small sized glaciers occur.

According to Brown et al. (1998), the area is characterized by the presence of discontinuous permafrost, although the area is now more characteristic of the sporadic permafrost zone (Johansson et al. 2011a, b). Permafrost occurs in the mountains above ~ 850 m a.s.l. on the northeast and east-facing slopes, and above 1100 m a.s.l. on the south-facing slopes (Ridefelt et al. 2008). At lower elevations, permafrost sporadically occurs in mires with ombrotrophic peat mounds (Johansson et al. 2006).

Soils are mostly composed of till, colluvium, and glaciofluvial deposits. More calcareous bedrock promoting higher nitrogen availability is found in the north-western parts of the study area and decreases towards the east, although some nutrient-rich areas are also found in the central part (Björk et al. 2007).

The fauna in the Abisko area is diverse and plays an important role in the ecosystem dynamics, with reindeer (*Rangifer tarandus*), moose (*Alces alces*), lemmings (*Lemmus lemmus*), voles (e.g. *Myodes rufocanus*) and some geometrid moth species (e.g. *Epirrita autumnata*) having a distinct impact on the vegetation dynamics of the area (Callaghan et al. 2013).

### Literature review

Five ecosystem components were explored in this study: local climate (temperature and precipitation), permafrost, hydrology, vegetation, and the carbon cycle. Long and short-term field and laboratory studies, modelling papers, and synthesis of multiple studies conducted in the Torneträsk area, were examined to identify (1) drivers (and their direct and indirect effects) that are changing the ecosystem components above, and (2) the underlying processes, or causal pathways, by which a driver could affect a specific ecosystem component. A total of 30 drivers and over 700 processes were identified (see Appendix S1).

### The expert assessment

Between May and August 2019, 27 leading scientists contributed to an Expert Assessment about ecosystem change in the Torneträsk area. The experts were selected based on their expertise in at least one of the five ecosystem components of interest, and on their previous work in the study area (for > 5 years, some up to > 50 years) (Appendix S3).

The Expert Assessment consisted of an online survey which was answered by each expert using the online platform surveygizmo (https://www.surveygizmo.com/). The methods employed in developing the survey were inspired from those designed by Sutherland et al. (2011), and were modified and adapted according to our objectives and needs.

The experts were asked to answer three questions for each of the 30 drivers explored (including both their direct and indirect impacts), and concerning the ecosystem component they had expertise in (Appendix S1). Question 1 asked them to rank (1–9) the importance of a given driver on the ecosystem component concerned, for the periods 2020–2040 (Question 1A) and 2040–2100 (Question 1B). Question 2 asked them to rank (1–9) how well studied are the potential future impacts of each driver on the ecosystem component concerned. Question 3 allowed the experts to provide self-reported expertise (1–5) for each particular driver. The experts had the option to suggest important studies that they believe need to be conducted in the future. The participants were provided with the following material (see Appendix S1): (i) general instructions; (ii) the findings of the literature review, and iii) a detailed example of how to answer the survey.

All responses belonging to the same group of experts were gathered and analysed together using the same methodology, which is described in detail in the supplementary material (Appendix S2). Responses for Question 1 (variable importance) were normalized on a 0–10 scale. The scores for Question 2 (variable awareness) were inverted in order to convert awareness into novelty, which is indicative of how new, or understudied, the ecosystem impacts of a given driver are. Subsequently, the novelty scores were normalized on a 0–10 scale. All responses for each variable (importance and novelty) were aggregated by averaging the normalized scores. In reporting results, responses with self-rated expertise of 1 (not familiar) were excluded. In this study, drivers presenting high importance (> 6) and high novelty (> 5) scores were considered research priorities.

## Results

In the Torneträsk region, 21 of the 30 drivers (including their direct and indirect effects) identified were ranked as the top ten most important drivers for at least one of the ecosystem components and study periods (Table 1). Air temperature was ranked as the most important driver for all ecosystem components and for both study periods, except for hydrology (where rainfall was top-ranked) and carbon cycle (where lake-ice duration was top-ranked for the period 2020–2040). Only air temperature, winter warming events, and snow cover were ranked in the top ten most important drivers for all the components and periods studied.

**Table 1:** Summary of the most important drivers (including their direct and indirect effects) (with mean importance estimates, on a 0–10 scale, calculated based on the experts’ responses from all groups; *n* = 5), and research priorities (identified by number of expert groups, on a 0–5 scale)

Most important drivers (mean importance estimates across all groups)		Research priorities (identified by number of expert groups)	
2020–2040	2040–2100	2020–2040	2040–2100
Air temperature (8.5)	Air temperature (8.9)	Winter warming events (5)	Winter warming events (5)
Snow cover (7.8)	Snow cover (8.2)	Evapotranspiration (3)	Evapotranspiration (5)
Winter warming events (7.3)	Rainfall (8)	Rainfall (3)	Rainfall (4)
Rainfall (7)	Winter warming events (7.4)	Snow cover (3)	Snow cover (3)
Snow depth (6.8)	Evapotranspiration (6.8)	Lake-ice duration (3)	Lake-ice duration (3)
Evapotranspiration (6.5)	Soil moisture (6.7)	Soil moisture (3)	Soil moisture (3)
Soil moisture (6.4)	Snow depth (6.5)	Droughts (2)	Drought (3)
Lake-ice duration (6.2)	Snow-water equivalent (6.2)	Snow-water equivalent (2)	Snow-water equivalent (2)
Snow-water equivalent (6)	Lake-ice duration (5.9)	Snow depth (2)	Snow depth (2)
Plant productivity (5.7)	Droughts (5.6)	River discharge – groundwater flow (2)	River discharge—groundwater flow (1)
River discharge—groundwater flow (5.7)	River discharge—groundwater flow (5.4)	Extreme rainfall events (1)	Extreme rainfall events (1)
Cloud cover (5.6)	Cloud cover (5.3)	Air temperature (1)	Air temperature (1)
Extreme rainfall events (5.4)	Dissolved organic carbon (5.2)	Plant productivity (1)	Plant productivity (1)
Droughts (5.1)	Extreme rainfall events (5.1)	Cloud cover (1)	Cloud cover (0)
Insect outbreaks (4.7)	Insect outbreaks (4.7)	Insect outbreaks (0)	Insect outbreaks (1)
Active layer thickness (4.7)	Active layer thickness (4.2)		
Reindeer herding (4.4)	Insect population (4)		
Insect population (3.4)	Plant productivity (3.9)		
Rodents population (3.2)	Rodents population (3.4)		
Dissolved organic carbon (2.9)	Black carbon (3.3)		
Black carbon (2)	Reindeer herding (2.6)		

A total of 15 drivers were identified as research priorities for at least one of the ecosystem components and periods included in the study (Table 1). Of these, only rainfall, evapotranspiration, and winter warming events were ranked as research priorities for all the components elicited, for at least one study period. Furthermore, winter warming events was the only driver ranked as a research priority for all components and time periods.

A summary of the important future studies suggested by the different groups of experts is available in the Supplementary Material (Appendix S4). The experts' estimates of importance and novelty, for the top 10 most important drivers for each ecosystem component, are summarized below and in Appendix S3.

### Local climate

**Fig. 2:**
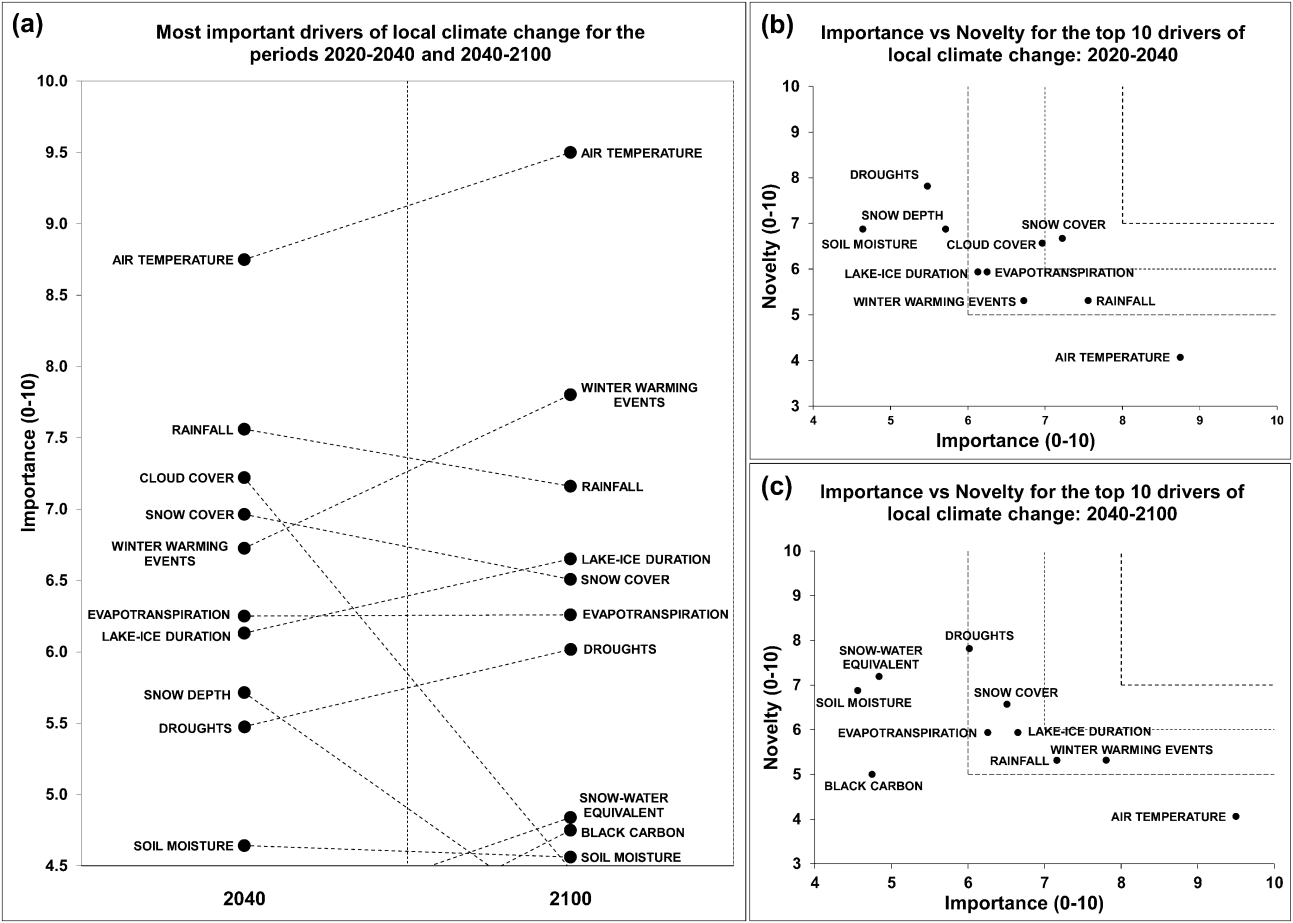
**a** The ten most important drivers of local climate change for the periods 2020–2040 and 2040–2100. **b**, **c** Future research priorities identified through importance vs novelty for the most important drivers of local climate change, for the periods 2020–2040 and 2040–2100, respectively

The relative importance of four drivers (air temperature, winter warming events, lake-ice duration, and droughts) increased over time (Fig. 2a and Appendix S3). On the contrary, large decreases in relative importance were observed for rainfall, snow cover, cloud cover, and snow depth. The changes in the relative importance of these drivers over time predicted by the experts resulted in changes in their scores and relative positions in the ranking, excluding cloud cover and snow depth, and incorporating snow water equivalent and black carbon in the top ten list for the period 2040–2100.

The research priorities identified for the period 2020–2040 (Fig. 2b) were snow cover, cloud cover, lake-ice duration, winter warming events, rainfall, and evapotranspiration. For the period 2040–2100, snow cover, lake-ice duration, evapotranspiration, rainfall, and winter warming events, were still perceived as important topics for further studies, in addition to droughts (Fig. 2c).

### Permafrost

**Fig. 3:**
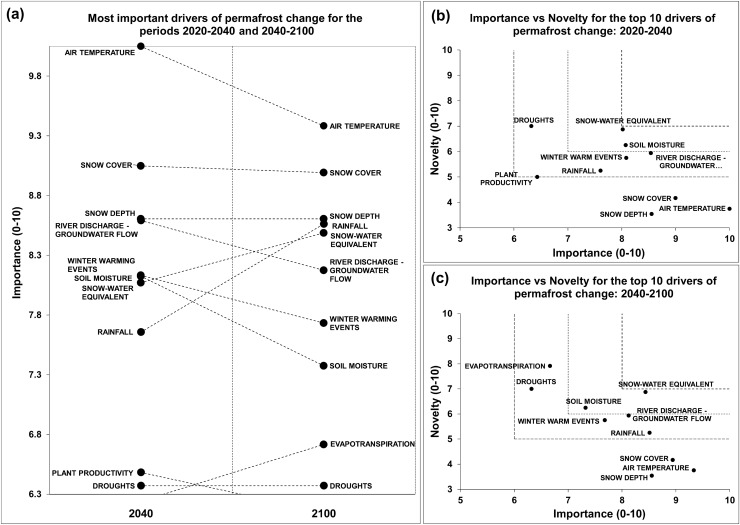
**a** The ten most important drivers of permafrost change for the periods 2020–2040 and 2040–2100. **b**, **c** Future research priorities identified through importance vs novelty for the most important drivers of permafrost change for the periods 2020–2040 and 2040–2100, respectively

The relative importance of all drivers decreased over time, except for rainfall, snow-water equivalent and evapotranspiration (Fig. 3a and Appendix S3). For the period 2040–2100, the top ten list of most important drivers excluded plant productivity, but included evapotranspiration.

For the period 2020–2040, snow water equivalent, droughts, soil moisture, river discharge and groundwater flow, winter warming events, and rainfall, were suggested as permafrost research priorities (Fig. 3b). All of these drivers were still perceived as priority research for the period 2040–2100, in addition to evapotranspiration (Fig. 3c).

### Hydrology

**Fig. 4:**
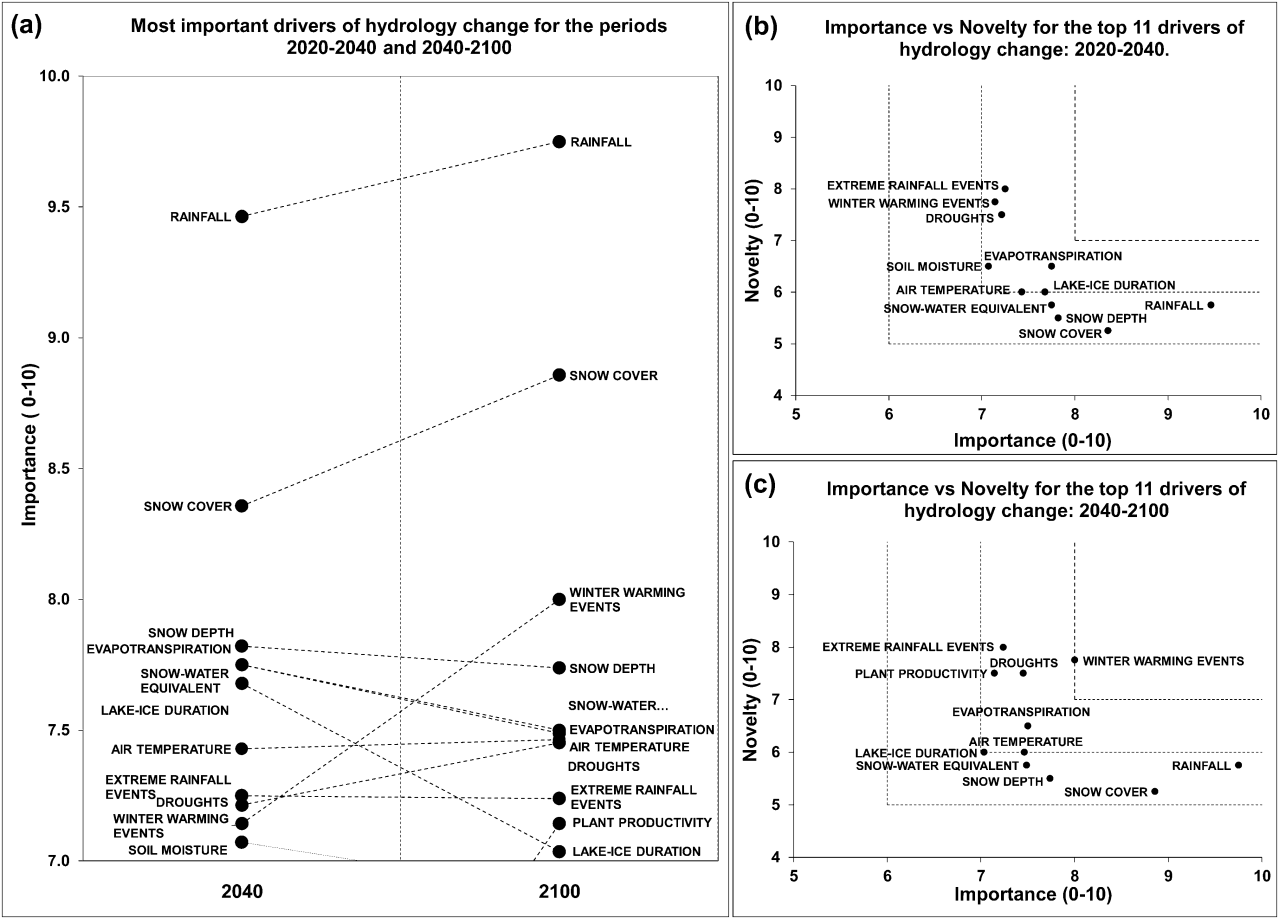
**a** The eleven most important drivers of hydrology change for the periods 2020–2040 and 2040–2100. **b**, **c** Future research priorities identified through importance vs novelty for the most important drivers of hydrology change for the periods 2020–2040 and 2040–2100, respectively

Given the particularly high importance and novelty scores assigned to a large number of hydrological drivers, we retained drivers presenting a mean importance score > 7 in the top list of important drivers (Fig. 4a and Appendix S3). The relative importance of four drivers (rainfall, snow cover, winter warming events and droughts) increased over time. On the contrary, substantial decreases are visible in the relative importance of snow depth, snow-water equivalent, lake-ice duration, and soil moisture, in 2040–2100. These changes resulted in the exclusion of soil moisture and the addition of plant productivity in the top 11 list of important drivers for the period 2040–2100.

For the period 2020–040, winter warming events, extreme rainfall events, droughts, evapotranspiration, lake-ice duration, air temperature, and soil moisture, were identified as hydrology research priorities (Fig. 4b). Of these drivers, only soil moisture was no longer perceived as a research priority for the period 2040–2100. In addition, plant productivity was included as a research priority (Fig. 4c).

### Vegetation

**Fig. 5:**
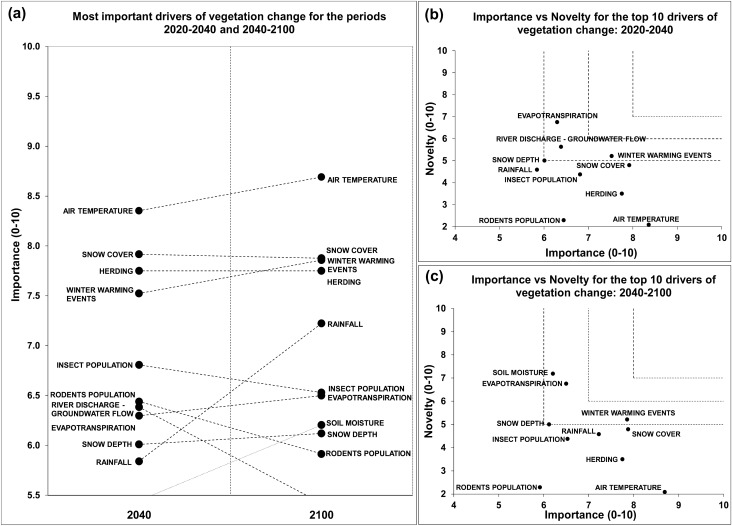
**a** The ten most important drivers of vegetation change for the periods 2020–2040 and 2040–2100. **b**, **c** Future research priorities identified through importance vs novelty for the most important drivers of vegetation change for the periods 2020–2040 and 2040–2100, respectively

Substantial increases over time were observed in the relative importance of air temperature, rainfall, winter warming events, and soil moisture (Fig. 5a and Appendix S3). In contrast, decreases were observed in the relative importance of insect population, rodent populations, river discharge, and groundwater flow. These changes resulted in the exclusion of river discharge and groundwater flow, and the incorporation of soil moisture in the top 10 list for the period 2040–2100.

The vegetation research priorities identified for the near future (2020–2040) were evapotranspiration, river discharge and groundwater flow, winter warming events, and snow depth (Fig. 5b). With regard to the period 2040–2100, evapotranspiration, winter warming events, and snow depth, remained as research priorities, in addition to soil moisture (Fig. 5c).

### Carbon cycle

**Fig. 6:**
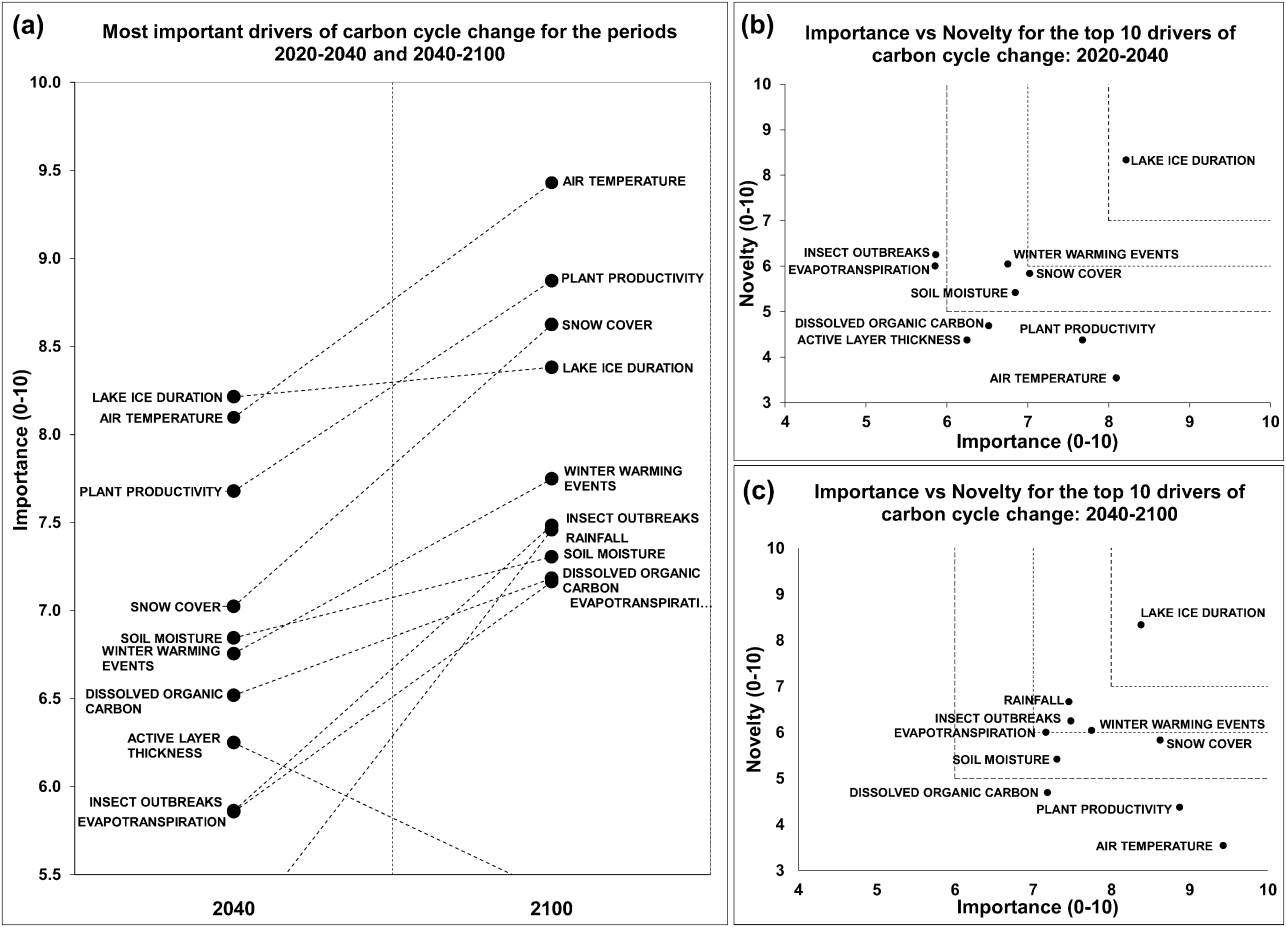
**a** The ten most important drivers of carbon cycle change for the periods 2020–2040 and 2040–2100. **b**, **c** Importance vs novelty for the most important drivers of cycle change for the periods 2020–2040 and 2040–2100, respectively

The mean estimates from all expert responses indicate a projected strong increase over time in the relative importance of all the top ten most important drivers, with the exception of active layer thickness, which was excluded from the top ten list for the period 2040–2100 (Fig. 6a and Appendix S3).

The drivers identified by the experts as research priorities for the period 2020–2040 are lake-ice duration, winter warming events, snow cover, and soil moisture (Fig. 6b). These four drivers, together with rainfall, insect outbreaks, and evapotranspiration, represent the carbon cycle research priorities for the period 2040–2100 (Fig. 6c).

## Research priorities and ways forward

In this study, the drivers (including their direct and indirect impacts) of ecosystem change in the Torneträsk area were ranked, and future research priorities were identified. In this section, we will focus on the top research priorities identified by at least three groups of experts (out of five; on local climate, permafrost, hydrology, vegetation, and the carbon cycle). These research priorities are deemed to be the most important elements that require particular focus to underpin more robust future predictions of ecosystem changes in the study area. We particularly highlight important interactions among the drivers that have hitherto been neglected in the area.

We propose further studies on each of these drivers according to the 3 M concept (Johansson et al. 2012), using monitoring (in-situ and remote sensing; including a better collaboration with the local and Indigenous Peoples to increase the observational power), manipulation experiments (to simulate changes in the current dynamics of the drivers and evaluate the resulting impacts on ecosystems), and finally modelling (to upscale the local findings). This has been further developed into a 4 M concept to recognize the end point of “management” (Callaghan *pers.comm*).

### Winter warming events

Direct and indirect effects of winter warming events on ecosystem change were identified as a research priority by all expert groups. In the study area, the frequency of winter warming events has been studied for the last century, showing a peak of events in the 1920s–30 s, and a stronger one during the last two decades (Vikhamar-Schuler et al. 2016). There are also a few studies on impacts of extreme winter warming events that sprung out of a collaboration with Indigenous Peoples, who had observed increasing ice layers in the snowpack after extreme winter warming events (Riseth et al. 2011). This studies show that winter warming events, mainly through altering the snow insulating effect and the plant available water in growing seasons, are a potential driver of the ‘browning’ of vegetation (declining biomass or productivity) recently observed in some parts of the Arctic (Phoenix and Bjerke, 2016). Bokhorst et al. (2009) observed a large decline (26%) in vegetation greenness (NDVI, normalized difference vegetation index) after the severe winter warming event during December 2007, although this damage was followed by a quick (within 2 year) recovery (Bokhorst et al. 2012). The impacts on vegetation growth and other ecosystem processes by winter warming events are likely to intensify in the scenario of more frequent and intense events predicted for the coming decades (Vikhamar-Schuler et al. 2016).

Till now, there are only a few studies available in the Arctic area focusing on the direct and indirect impacts of extreme winter warming events on snow duration and properties, albedo, permafrost, microbial activity, vegetation dynamics, herbivore populations and biodiversity (e.g. Schimel et al. 2004; Callaghan et al. 2011; Sokolov et al. 2016; Barrere et al. 2018; Treharne et al. 2019). The impacts of these events still remain largely uncertain for most of the Arctic, including our study area. The most important research questions identified in this study (Supplementary material S4) cover most of the topics above, and include research questions such as “*What is the impact of increasing extreme winter warming events on mortality of animals and plants, and the capacity to open space for invasive species?”,* “*How do different snow conditions and vegetation characteristics influence the impacts of winter warming events on ground temperatures*?”, and “*What is the impact of increasing extreme winter warming events on stream flow, and how does this affect hydropower?*”.

In order to obtain the information needed to improve predictive models and facilitate future management, we suggest to (1) improve the current monitoring system by (i) developing remote sensing techniques capable of quantifying changes in snowpack properties at relevant spatial and temporal scales, and (ii) implementing high-resolution monitoring of stream flow, including winter time, (2) perform manipulation studies to investigate impacts of winter warming events on (i) land cover types other than dwarf shrub heathland (which has been covered by e.g. Bokhorst et al. (2010)), and (ii) on the snow thermal conductivity and ground temperatures across a latitudinal gradient, and under different snow and vegetation conditions, (3) conduct manipulation studies simulating more intense and frequent winter warming events, as well as co-occurring winter warming and other extreme events, such as severe droughts and insect outbreaks, to evaluate the resulting responses of vegetation, ground temperatures and the carbon cycle, and (4) improve the representation of snow-related processes such as snowmelt, rain water percolation and refreeze in the snowpack, and the insulating capacity of snow, in ecosystem models.

### Evapotranspiration

Direct and indirect effects of evapotranspiration on ecosystem change were identified as a research priority by all expert groups. There are no studies on the direct and indirect impacts of evapotranspiration on ecosystems in the study area. Annual mean evaporation in northern Sweden is projected to increase by between 0.1 and 0.4 mm day^−1^ by 2100 (IPCC 2013). Future changes in the water balance, however, will also depend on changes in precipitation, wind speed, and vegetation type and distribution (Allen et al. 1994). Since the increases in annual precipitation for the twenty-first century are largely expected in winter, when evapotranspiration rates are low (IPCC 2013), it is likely that, under a future warmer climate, soil moisture will decrease in summer. Nevertheless, these predictions (and hence the resulting consequences for ecosystems) are highly uncertain.

Studies on the direct and indirect impacts of evapotranspiration on local and regional air temperature (e.g. Ban-Weiss et al. 2011; Pearson et al. 2013) and on soil properties (soil moisture, thermal conductivity, and temperature) (e.g. Lawrence and Swensson 2011), exist from a few Arctic locations, but studies on the resulting impacts on plant productivity and microbial activity are lacking. All of these processes, in turn, require further attention in the study area. The most important research questions suggested by the experts (Supplementary material S4) cover most of these topics and include research questions such as “*What is the potential for shifts in evapotranspiration to cause water deficits in contrasting landscape positions and on different timescales?,* and *“What are the impacts of hydrological regime shifts on (i) vegetation dynamics, (ii) ground temperatures, (iii) microbial activity and soil organic carbon decomposition, (iv) water flow, and the transport, delivery and fate of dissolved organics, and (v) the carbon balance?”.*

A suggested way forward in the study area is to (1) implement continuous evapotranspiration monitoring, and expand and sustain the current precipitation monitoring network, to understand the changes in the water balance over the study region, (2) conduct manipulation studies to quantify ecosystem responses (e.g. plant-specific responses, soil temperature and moisture, soil microbial activity, and water flow and terrestrially derived compounds) to scenarios of increased evapotranspiration, and (3) improve the representation of the evapotranspiration-climate interactions in models.

### Rainfall

Direct and indirect effects of rainfall on ecosystem change were identified as a research priority by four of the five expert groups: all but vegetation experts. In the study area, an increase in rainfall has occurred especially since 1980 (Callaghan et al. 2010), with a dramatic increase in the magnitude of extreme rainfall events over the past century that have caused damage in infrastructures and destabilized mountain slopes (Jonasson et al. 2012). Impacts of increasing rainfall, such as the increased transport of dissolved organic matter (DOM) in water bodies, have been studied in the Torneträsk region (e.g. Kokfelt et al. 2009; Giesler et al. 2014). The increased DOM concentration in waterbodies may be enhanced in the long term due to permafrost thawing (e.g. Olefeldt and Roulet, 2012), and the larger amounts of plant biomass (e.g. Tang et al. 2018.). Karlsson et al. (2010) suggested that future increases in summer precipitation and loss of sporadic permafrost could lead to a net release of carbon to the atmosphere through respiration. The field manipulation studies that artificially increased summer precipitation do not show any significant impacts on the growth of vascular plants (e.g. Karlsson, 1985; Parsons et al. 1994; Keuper et al. 2012), but indicate that bryophytes may benefit from increased precipitation (Phoenix et al. 2001), which may increase ecosystem productivity given their substantial role in C cycling at high latitudes (Street et al. 2013).

Even if rainfall has been studied in the Torneträsk region for more than a century, different research gaps on the direct and indirect effects of rainfall on ecosystems needs to be addressed. As explained earlier, recent studies suggest that the future increase in summer rainfall is not likely to compensate the greater evapotranspiration water losses in the Torneträsk area (IPCC 2013). This imbalance can potentially result in reduced soil moisture, water flow, and organic matter transport, as well as altered vegetation and permafrost dynamics, which need further investigation in the area. Most of these topics were identified among the current research gaps suggested by the experts (Supplementary material S4), in addition to research questions such as “*What is the spatial and temporal effects of the rainfall-induced increases in evapotranspiration and vegetation productivity on the surface energy balance (latent heat and albedo effects)?*” and “*What will be the net effect of future changes in rainfall on the hydrologic system, and what impacts will it have on (i) the transport, delivery and fate of terrestrial carbon, (ii) plant productivity, (iii) permafrost dynamics, (iv) the carbon cycle?”.*

A suggested way forward in the study area is to (1) build a more robust and sustained precipitation and evapotranspiration monitoring network, to help reducing the uncertainties on the timing and magnitude of future changes in the water balance, (2) evaluate the impacts of increased rainfall on mountain permafrost, and (3) perform manipulation studies to assess the vegetation/permafrost/carbon cycle response to, in contrast to what has been assumed to date, a decrease in soil moisture.

### Snow cover

Direct and indirect effects of snow cover on ecosystem change were identified as a research priority by three of the five expert groups: local climate, hydrology, and carbon cycle expert groups. In the Torneträsk area, mean snow depth has doubled over the 20th Century (Kohler et al. 2006), whilst snow cover duration has decreased significantly at both high and low elevations between 1978 and 2007 (0.1 and 0.12 week year^−1^; Andrews et al. 2011). In addition, a long-term (49-year) record of snow profile stratigraphy showed increases in hard snow layers, and changes in snow hardness and dryness during early winter and spring (Johansson C. et al. 2011), mostly due to more intense and frequent abrupt winter temperature fluctuations recently occurring in the area (Vikhamar-Schuler et al. 2016). These changes in snow cover and properties have important consequences for arctic ecosystems and societies (Callaghan et al. 2011). The field snow addition by snowfence have resulted in substantial increases in ground temperature, active layer thickness, and growth and distribution of graminoids, in a peat plateau with permafrost in Torneträsk area (Johansson et al. 2013). Other studies have observed substantial vegetation frost-damage in response to warming-induced changes in snow properties (e.g. Bokhorst et al. 2009). Projections for the Torneträsk area indicate strong reductions in snow depth and cover over the twenty-first century (Brown et al. 2017), which may exacerbate the related impacts.

Even though a growing body of literature on the Arctic winter climatic change have shed light on the ecosystem responses to changes in snow properties (see Wipf and Rixen 2010; Cooper 2014; Bokhorst et al. 2016, and references therein), further advances in snow monitoring and modelling are required, and studies on the impacts of snow changes on ecosystem processes, such as the surface energy budget, seasonal biological and hydrological responses, and trophic-level interactions, deserve a greater attention in the study area. The most important research questions identified in this study (Supplementary material S4) cover most of those topics and include research questions such as “*What is the spatial distribution of snow depth and stratigraphy in the study area, and how does it affect soil moisture, soil temperatures, and soil microbial activity?*” and “*What is the balance between shorter snow-pack periods and anticipated greater snowfall, and how does it affect the timing of snowmelt and the related hydrological and stream ecological processes?”.*

A suggested way forward in the study area is to address major gaps that impede performing better projections of changes in snow properties: (1) monitoring gaps, by (i) extending the number of human-based and automatic measurements of snow properties, (ii) including other sources of knowledge, such as traditional ecological knowledge (TEK) (Riseth et al. 2011), and (iii) developing and improving remote sensing techniques capable of retrieving accurate data on snow properties at relevant spatial and temporal scales; (2) experimental gaps, by performing studies of the impacts of a changing snow cover on (i) biological activities in autumn, (ii) trophic-level interactions, and (iii) microbial activity and the decomposition of organic matter in soils; (3) modelling gaps, by improving the representation of arctic snow cover, and the representation of snow-related processes (e.g. snowmelt, snow albedo, snow insulating capacity, and snow-wind and snow-freshwater ice interactions) in models.

### Lake-ice duration

Direct and indirect effects of lake-ice duration on ecosystem change were identified as a research priority by three of the five expert groups: local climate, hydrology, and carbon cycle expert groups. Lake-ice duration has decreased substantially in the study area during the twentieth century, as observed in Lake Torneträsk (47 days decline during the twentieth century; Callaghan et al. 2010). Different studies have investigated the impacts of the declining lake-ice duration on ecosystems in the study area, including the effects on air temperature in the adjacent areas (Yang et al. 2011), lake primary productivity (Karlsson et al. 2009), and CO_2_ (Denfeld et al. 2016) and CH_4_ (Wik et al. 2014) emissions. These impacts are likely to intensify with the projected further shortening of lake-ice duration in the area (Prowse et al. 2012).

Studies on future lake-ice dynamics, and potential direct and indirect impacts on ecosystem processes such as aquatic primary productivity (e.g. Rühland et al. 2015), emissions of CO_2_ and CH_4_ (e.g. Wik et al. 2014; Denfeld et al. 2016), and the climate (e.g. Brown and Duguay 2010), exist from other locations across the Arctic. However, as identified in the expert elaborations (Supplementary material S4), there is a great need for accurate estimates of future lake-ice decline rates in the study area, and investigations on the resulting implications for the hydrologic system and the carbon cycle. In addition, the experts suggested other important research questions such as “*What are the future changes in lake-ice duration and its effects on the local climate of the Torneträsk area?*”, and “*What are the effects on stratification and water circulation patterns, and their implications for carbon cycling (that could be profound in a water body the size of Torneträsk)?”.*

A suggested way forward in the study area is to (1) perform modelling studies to obtain accurate estimates of the future lake-ice decline rates, (2) integrate the future lake-ice dynamics and the resulting climate-hydrology-carbon cycle interactions into fine-scale models, in order to better asses the direct and indirect impacts of changing lake-ice conditions on (i) the climate, vegetation, ground temperatures, and the carbon cycle, on the adjacent ecosystems, and (ii) the water and sediment temperature, light penetration, water runoff, input of organic matter, primary productivity, and C fluxes, in water bodies.

### Soil moisture

Direct and indirect effects of soil moisture on ecosystem change were identified as a research priority by three of the five expert groups: local climate, hydrology, and carbon cycle expert groups. As discussed earlier, projections indicate a substantial decrease in soil moisture through the twenty-first century, especially during summer (IPCC 2013). These projections, however, remain highly uncertain due to the unknown balance between increasing evapotranspiration and precipitation, and the changing vegetation cover (IPCC 2013). As explained for rainfall above, there are no studies that investigated plant responses to reduced soil moisture in the Torneträsk area. In addition, studies evaluating the effects of decreasing soil moisture on permafrost and the hydrologic system are, to our knowledge, lacking in the study area.

The key role of soil moisture in modulating relevant ecosystem processes and parameters, such as ground temperature, decomposition rates of organic matter, and the form and magnitude of soil carbon emissions, is well recognized in the literature (e.g. Lin 1980; Oertel et al. 2016). However, at a local scale, near-surface soil moisture depends on several processes (e.g. infiltration, drainage, and active layer thickening), weather conditions (e.g. wind speed and radiation), and geophysical properties (e.g. surface roughness, soil texture, and permeability), for which we lack understanding at relevant spatial and temporal scales. This makes changes in soil moisture heterogeneous and challenging to predict across the landscape. Recent efforts have focused on retrieving fine-resolution satellite soil moisture data from different Arctic locations, and its assimilation in models (e.g. Watts et al. 2014; Zwieback et al. 2019). Yet, these methodologies still have major limitations, such as spatial and temporal coverage, and their coarse resolution. The most important research questions identified in this study (Supplementary material S4) cover most of the above-mentioned topics, and include research questions such as “*What are the spatial and temporal patterns of soil moisture conditions in the Torneträsk area?*” and “*What are the impacts of changes in soil moisture for ground temperatures and primary productivity?”.*

A suggested way forward in the study area is to (1) improve the monitoring system, by (i) by developing an extensive and continuous soil moisture monitoring programme, with special focus on underrepresented areas, such as mountainous terrain, and (ii) developing and improving remote sensing techniques to acquire frequent and spatially extended high-resolution soil moisture data, supported by the higher number of in-situ measurements, (2) perform manipulation studies on vegetation, permafrost, and the carbon cycle, in contrasting landscape positions and locations, assuming a future decrease in soil moisture, and (3) reduce uncertainties in the predictions of future changes in temperature and precipitation to obtain more accurate predictions of the future water balance,

### Droughts

Direct and indirect effects of droughts on ecosystem change were identified as a research priority by three of the five expert groups: local climate, permafrost and hydrology expert groups. Droughts are not causing major impacts on lowland ecosystems in the Torneträsk area at present (Bjerke et al. 2014), which has led to a scarce number of studies in the area. In contrast, numerous studies evaluating the effects of droughts on ecosystem processes such as plant productivity (e.g. Lotsch et al. 2005), soil moisture and ground water (e.g. Okkonen et al. 2010), the carbon cycling (e.g. Reichstein et al. 2013), fires (e.g. Kasischke and Turetsky 2006), soil respiration (e.g. Sowerby et al. 2008), and permafrost dynamics (e.g. Fisher et al. 2016), exist from several Arctic areas.

The current circumstances in the Torneträsk area may change in the future as droughts may become more frequent and intense in the Arctic (IPCC 2013). Some ongoing studies point towards this direction: the last major heatwave in the Torneträsk area, in July 2018 (3rd warmest July since 1913, with mean daily air temperatures up to 23.3 °C) (ANS, 2020. Meteorological data from the Abisko Observatory, monthly mean 2000–01-01–2019–12-31), and the associated decrease in soil moisture, might have reduced maximum active layer thickness in areas of permafrost thawing relative to the previous year, which experienced a colder spring and summer (Johansson M. et al., in prep); warming is projected to replace birch forest areas by more fire-vulnerable pine species in some areas (Wolf et al. 2008). Hence, the impacts of droughts clearly deserve further research focus in the Torneträsk area. Most of the topics mentioned above have been identified in the experts’ written elaborations (Supplementary material S4), in addition to research questions such as *“What is the relation between the Scandinavian (high-pressure) blocking of the jet stream, and the local meteorology in the study area, and how will its frequency change in the future?”,* and “*What are the impacts of droughts on stream ecology and biogeochemistry?”.*

A suggested way forward in the study area is to (1) perform field manipulation studies to investigate (i) the plant-specific responses to more severe and frequent droughts, and (ii) the impacts of droughts on soil temperature and soil moisture in contrasting landscape positions and land cover types, and the resulting effect on soil respiration, (2) investigate, through monitoring and modelling, the impact of droughts (i) on lowland and mountain permafrost, and (ii) on streamflow and water chemistry, aquatic primary productivity, and C fluxes from water bodies, (3) conduct modelling studies to assess how long-term vegetation changes, together with the occurrence of severe droughts, may favour fire disturbances, and (4) integrate and upscale findings from points 1–3 in models, to obtain a comprehensive assessment of the overall impact of droughts on the carbon cycle at a landscape scale.

## Conclusions

This expert evaluation of the importance and novelty of multiple ecosystem drivers in two future periods provides a comprehensive assessment of the current state of knowledge, and gives insights on research priorities surrounding ecosystem change in the Torneträsk area. The results further reveal the important knowledge gaps regarding the potential future impacts of different drivers. The most important research priorities identified include investigations of the current and potential effects on ecosystems brought on by altered frequency and intensity of winter warming events, evapotranspiration rates, rainfall, duration of snow cover and lake-ice, changed soil moisture, and droughts.

Because of the great complexity of arctic systems, a good understanding of the multiple causes of ecosystem change and the interactions between systems can often be best captured by focusing on a single location. The Torneträsk area, with its relatively small size, its great biological, meteorological and geomorphological diversity, and its unique datasets, is therefore suitable for such comprehensive analysis, and represents a microcosm of the Subarctic and the rapidly-transforming arctic ecosystems. The understanding obtained in this area can, despite the great diversity of arctic ecosystems, be applied in other arctic areas, and inform research efforts that, combined, can help improve future predictions. These predictions will provide local stakeholders with essential detailed information that will aid the development of mitigation plans and adaptation strategies.

## Electronic supplementary material

Below is the link to the electronic supplementary material.

Supplementary file 1 (PDF 4032 kb)
