# Supplementary material for: The missing pieces for better future predictions in subarctic ecosystems: A Torneträsk case study
Source: Ambio. 2020 Sep 13;50(2):375–92. doi: 10.1007/s13280-020-01381-1 (PMC7782653; doi:10.1007/s13280-020-01381-1)
Supplement: Supplementary file 1 — (PDF 4032 kb) [file 13280_2020_1381_MOESM1_ESM.pdf]

Ambio

Electronic Supplementary Material

This supplementary material has not been peer reviewed

Title: **The missing pieces for better future predictions in subarctic ecosystems: a Torneträsk case study**

Didac Pascual , Jonas Åkerman, Marina Becher, Terry V. Callaghan, Torben R. Christensen, Ellen Dorrepaal, Urban Emanuelsson, Reiner Giesler, Dan Hammarlund, Edward Hanna, Annika Hofgaard, Hongxiao Jin, Cecilia Johansson, Christer Jonasson, Jonatan Klaminder, Jan Karlsson, Erik Lundin, Anders Michelsen, David Olefeldt, Andreas Persson, Gareth K. Phoenix, Zofia Raczkowska, Riikka Rinnan, Lena Ström, Jing Tang, Ruth K. Varner, Philip Wookey, Margareta Johansson

Appendix S1. Expert assessment survey protocol.

General instructions

The aim of this study is to identify and rank drivers that directly or indirectly will contribute to ecosystem changes in the Torneträsk catchment in the short term (present – 2040) and long term (2040 – 2100). Moreover, we aim to identify and rank the drivers that are important but have received little attention so far.

The five ecosystem components included in this study are the **local climate, permafrost, hydrology, vegetation** and **carbon cycle**. Through an exhaustive literature review, different drivers affecting these components were identified and grouped in different categories according to their nature: *atmosphere, cryosphere, hydrosphere, extreme abiotic events, biosphere, extreme biotic events,* and *human activities*. Each of these drivers have undergone or are projected to undergo a shift in their dynamics as a result of changes in climate and/or human behaviour, which in turn might have an impact on the ecosystem components (see Appendix II for further information about the identified drivers).

You are invited to contribute to the ______ component. The survey is composed of eight sections. The first *seven sections* are each dedicated to one group of drivers. In each of these sections we kindly ask you to answer the same *three questions* for each of the drivers included in that section:

- *Question 1* will ask you to rank the importance of a given driver on the ecosystem component concerned, for the period 2020-2040 (Question 1A) and for the period 2040-2100 (Question 1B).

- *Question 2* will ask you to rank how well studied are the potential future impacts of each driver on the ecosystem component concerned.

- *Question 3* will ask you to rank your expertise in each particular driver.

The final (eighth) section will ask you about final suggestions and thoughts, as well as personal data that will only be used for the purposes of this study.
The sections will be randomized in order to counteract the possibility of scoring fatigue and other unconscious issues during the scoring process.

If you want to stop completing the survey and continue later, you can use the *Save & Continue* feature at the top of the survey's page. This feature gives you the option to enter your email address to receive a link to return to your survey later.

To complete the survey, you are encouraged to make use of all knowledge, information, literature, models, advice, beliefs and 'gut feelings' available to you.

Given your great expertise in the topic and your great familiarity with the study area, we do not provide any general information prior to the completion of the survey. The familiarity with the study area is essential for this matter because the peculiarities of the Torneträsk catchment (climatic and geomorphological setting, distance from the lake, differences in slope or orientation, vegetation composition and distribution, etc) can significantly influence the ecosystem response to a given change, potentially leading to a different response than you would expect in other sub-arctic settings. These **peculiarities must be taken into account** when completing the survey.

A detailed example is attached below. Please keep in mind that **generalizations are needed**. Please don't hesitate to contact us if you have any questions or comments.

Thank you very much for your contribution to this study!

Figures and Tables

In order to support your reasoning, please find below a *Figure* showing the geographical extent of the study area, and 7 *Tables (*one per each group of drivers), per each component, which gather the different direct and indirect processes triggered by a change in each driver that can have an impact on the ecosystem component concerned. We encourage you to use this information taking into account the following considerations:

- The Tables **are not complete and do not include all the drivers and processes** affecting the ecosystem components. If considered convenient, you are allowed to suggest other drivers and processes of relevance in the sub-questions created for this purpose.

- The Tables present **dynamics and processes that have been generalized,** being aware that in reality they are more complex. At this stage of the study, generalizations are needed to reduce complexity, visualize the dominant processes and impacts, and obtain the results pursued.

- The processes presented in the Table **are not ranked nor sorted** according to any criteria.

- The ***Font colour*** **(red or blue)** applied to each process **indicate the expected future impact** that it exerts on the ecosystem component concerned (where red indicates enhancement or increase, and blue indicates diminution or decrease). ***Grey*** is applied when the expected impact cannot be defined as an enhancement or diminution.

- In the Tables, **the number of processes within each box sharing *Font colour* do not reflect the dominant effect** that a driver exerts on the ecosystem component concerned.

Figure S1. *Study area.*

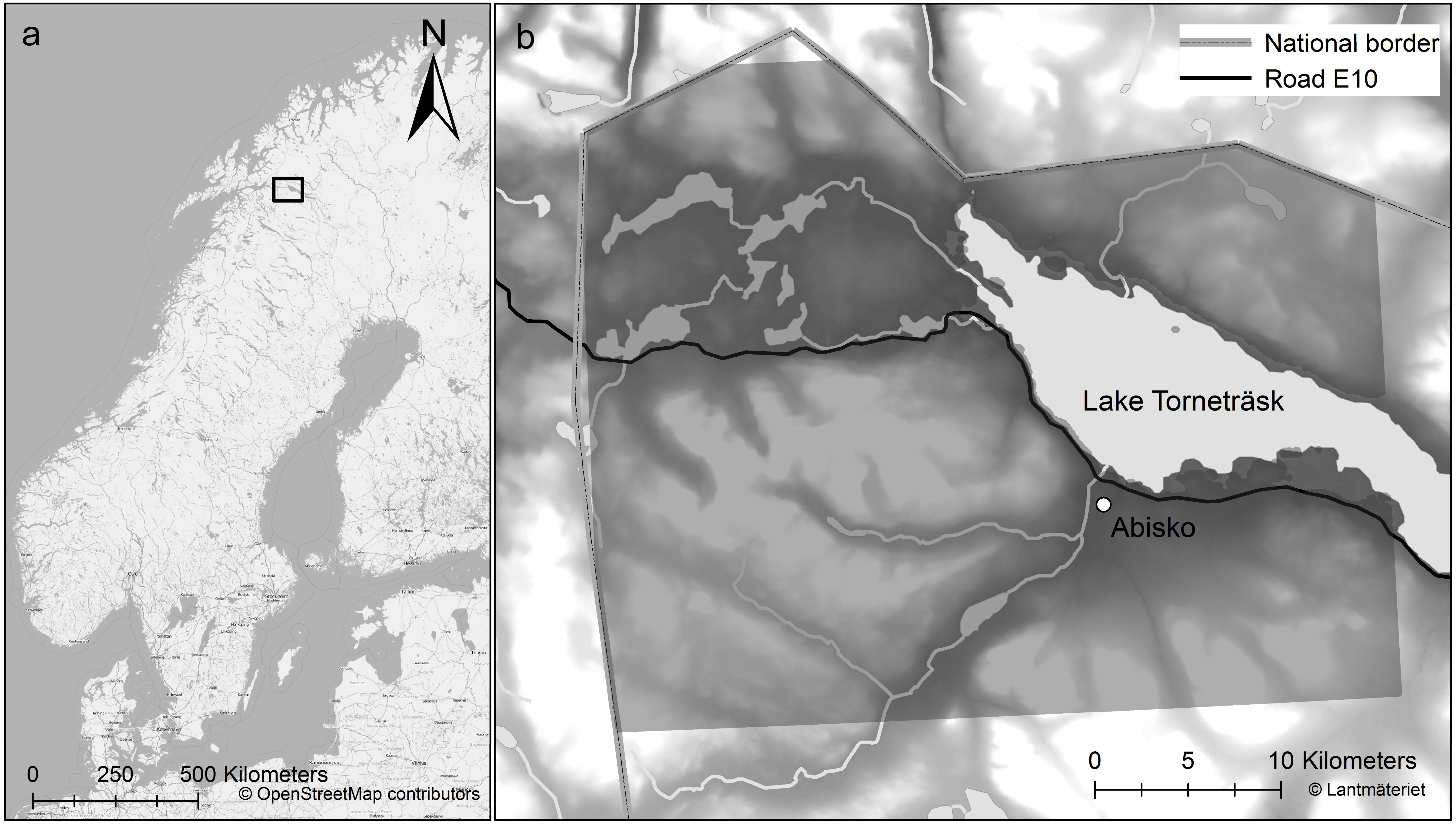

Ecosystem component: Local Climate

Table S1. *Atmospheric drivers.*

Ecosystem Component	**Atmospheric drivers**					
	**Increasing air temperature**	**Increasing rainfall**	**Increasing ET**	**Decreasing UV-B radiation**	**Increasing Cloud cover**	**Increasing atmospheric CO_2 concentrations**
**Local Climate**	• + Air temperature → - snow and lake ice season duration → - albedo → + air temperature<br>• + Air temperature → + forest/shrub → - albedo → + air temperature<br>• + Air temperature → + ET→ + atmospheric water vapor → + GH effect → + air temperature<br>• + Air temperature → taller vegetation → + vegetation masking → - snow cover duration → + air temperature<br>• + Air temperature → + ET→ + latent heat absorbed and + cloud cover → - air temperature<br>• + Air temperature → + forest/shrub → + ET → - air temperature<br>• + Air temperature → + BVOC release → + SOA → + albedo and + cloud cover → - air temperature	• + Rainfall → + plant productivity → + vegetation cover and density → - albedo → + air temperature<br>• + Rainfall → + ET→ + atmospheric water vapor → + GH effect → + air temperature<br>• + Rainfall → taller vegetation → + vegetation masking → - snow cover duration → + air temperature<br>• + Rainfall → + soil moisture and plant productivity → + ET → + latent heat absorbed and cloud cover → - air temperature<br>• + Rainfall → + plant productivity → + vegetation cover and density → + BVOC → + SOA → + albedo and + cloud cover → - air temperature	• + ET → + atmospheric water vapor → + GH effect → + air temperature<br>• + ET → + latent heat absorbed → - air temperature<br>• + ET → + water vapor → + cloud formation → - air temperature<br>• + ET → + cloud cover → + precipitation	• - UV-B radiation → + photosynthesis → + vegetation cover and density → - albedo → + air temperature	• + Cloud cover → overall – air temperature	• + Atmospheric CO_2 → + CO_2 fertilization → + plant productivity → + vegetation cover and density → - albedo → + air temperature<br>• + Atmospheric CO_2 → + vegetation cover and density → + ET → + atmospheric water vapor → + air temperature<br>• + Atmospheric CO2 → closure of stomatal pores → - ET → - latent heat absorbed and – cloud cover → + air temperature<br>• + Atmospheric CO2 → taller vegetation → + vegetation masking → - snow cover duration → + air temperature<br>• + Atmospheric CO2 → closure of stomatal pores → - ET → - atmospheric water vapor → - air temperature<br>• + Atmospheric CO_2 → + CO_2 fertilization → + plant productivity → + vegetation cover and density → + ET and + cloud cover → - air temperature<br>• + Atmospheric CO_2 → + CO_2 fertilization → + vegetation cover → + BVOC → + SOA → + albedo and + cloud cover → - air temperature

BVOC	Biogenic Volatile Organic Compound
CO_2	Carbon dioxide
ET	Evapotranspiration
GH	Greenhouse
SOA	Secondary organic aerosol
UV-B	Ultraviolet B

List of abbreviations used in Table S1.

Table S2. *Cryospheric drivers*

Ecosystem Component	**Cryospheric drivers**				
	**Decreasing snow cover duration**	**Increasing snow depth**	**Increasing active layer thickness**	**Decreasing lake ice duration**	**Decreasing glacier extent**
**Local Climate**	• - Snow cover duration → - albedo → + air temperature<br>• - Snow cover duration → overall + plant productivity → + vegetation cover and density → - albedo → + air temperature<br>• - Snow cover duration → - summer snowmelt and – ET → - latent heat absorbed → + air temperature<br>• - Snow cover duration → + vegetation cover and density → + ET → + atmospheric water vapor → + GH effect → + air temperature<br>• - Snow cover duration → + vegetation cover and density → + ET → + cloud cover and latent heat absorbed → - air temperature<br>• - Snow cover duration → - summer snowmelt and – ET → - atmospheric water vapor → - GH effect → - air temperature<br>• - Snow cover duration → + heat and particulate release → - stable boundary layer → + mid-level cloud formation → - air temperature	• + Snow depth → + ground temperature → + plant productivity → + vegetation cover and density → - albedo → + air temperature<br>• + Snow depth → + vegetation cover and density → + ET → + atmospheric water vapor → + GH effect → + air temperature<br>• + Snow depth → + snow on lake-ice → + insulation → - ice thickness → - lake-ice duration → - albedo → + air temperature<br>• + Snow depth → + vegetation cover and density → + ET → + cloud cover and latent heat absorbed → - air temperature<br>• + Snow depth → + SWE → + latent heat absorbed during snow melt → - air temperature.	• + Active layer thickness → + ground temperature → + carbon release to atmosphere → + air temperature<br>• + Active layer thickness → + ground subsidence → + thermokarst formation → - albedo → + air temperature	• - Lake ice duration → - albedo and + atmospheric. water vapor → + air temperature<br>• - Lake ice duration → + atmospheric water vapor → + precipitation<br>• - Lake ice duration → + ET → + cloud cover and + latent heat absorbed → - air temperature effect	• - Glacier extent → - albedo → + air temperature

ET	Evapotranspiration
GH	Greenhouse
SWE	Snow water equivalent

List of abbreviations used in Table S2.

Table S3. Hydrospheric drivers

Ecosystem Component	**Hydrospheric drivers**			
	**Decreasing soil moisture**	**Increasing SWE**	**Increasing RD and GWF**	**Increasing lake/river DOC concentrations**
**Local Climate**	• - Soil moisture → - ET → - latent heat absorbed → + air temperature<br>• - Soil moisture → - ET → - cloud cover → + air temperature<br>• - Soil moisture → - plant productivity → - vegetation cover and density → - BVOC → - SOA and cloud cover → + air temperature<br>• - Soil moisture → - ET → - atmospheric water vapor → - GH effect → - air temperature<br>• - Soil moisture → - plant productivity → - forest and shrub cover and density → + albedo → - air temperature<br>• - Soil moisture → - plant productivity → taller vegetation → - vegetation masking → + snow cover duration → - air temperature	• + SWE → + ground temperature and + soil moisture → + vegetation cover and density → - albedo → + air temperature<br>• + SWE → + vegetation cover and density → + ET → + atmospheric water vapor → + GH effect → + air temperature<br>• + SWE → → taller vegetation → + vegetation masking → - snow cover duration → + air temperature<br>• + SWE → + ground temperature and + soil moisture → + vegetation cover and density → + ET and + cloud cover → - air temperature<br>• + SWE → + snow melt and sublimation, and + ET → + latent heat absorbed → - air temperature	No major local effects known.	No major local effects known.

BVOC	Biogenic Volatile Organic Compound
ET	Evapotranspiration
GWF	Ground water flow
RD	River discharge
GH	Greenhouse
SOA	Secondary organic aerosol

List of abbreviations used in Table S3.

Table S4. *Extreme abiotic events as drivers of ecosystem change*

Ecosystem Components	**Extreme abiotic events**			
	**Increasing WWE**	**Increasing extreme rainfall events**	**Increasing Droughts**	**Increasing wild-fires**
**Local Climate**	• + WWE → + ROS and WSE → - SWE → - snow cover duration → - albedo → + air temperature<br>• + WWE → + ROS and WSE → + vegetation damage → - shrub cover and density → + albedo → - air temperature<br>• + WWE → + ROS and WSE → - snow on lake-ice → - insulation → + ice thickness → + lake-ice season → + albedo → - air temperature<br>• + WWE → + ROS and WSE → + vegetation damage → changes in albedo → changes in air temperature	No major local effects known.	• + Droughts → - ET → - latent heat absorbed → + air temperature<br>• + Droughts → - ET → - atmospheric water vapor → - GH effect → - air temperature<br>• + Droughts → - vegetation cover and density → - surface albedo → - air temperature	• + Fire → - vegetation cover and density → - BVOC (after the fire-induced boost of BVOC) → - SOA → - albedo and - cloud cover → + air temperature<br>• + Fire → - vegetation cover and density → - ET → - latent heat absorbed → + air temperature<br>• + Fire → - forest and shrub cover → + surface albedo and – atmospheric water vapor → - air temperature<br>• + Fire → - vegetation cover and density → - vegetation masking → + snow cover duration → - air temperature

BVOC	Biogenic Volatile Organic Compound
ET	Evapotranspiration
GH	Greenhouse
ROS	Rain on snow
SOA	Secondary organic aerosol
WSE	Winter snowmelt event
WWE	Winter warming event

List of abbreviations used in Table S4.

Table S5. *Biospheric drivers*

Ecosystem Component	**Biosphere**				
	**Increasing Plant Productivity**	**Increasing BVOC emissions**	**Increasing large herbivores**	**Decreasing rodents population**	**Increasing insects population**
**Local Climate**	• + Plant productivity → + shrub and forest cover → - albedo and + atmospheric water vapor → + air temperature<br>• + Plant productivity → + shrub and forest cover → + snow masking → - snow cover duration → - albedo → + air temperature<br>• + Plant productivity → + vegetation cover and density → + ET → + latent heat absorbed and + cloud cover → - air temperature<br>• + Plant productivity → + vegetation cover and density → + BVOC → + SOA → + albedo and + cloud cover → - air temperature	• + BVOC → + (SOA) → + albedo → - air temperature<br>• + BVOC → + SOA → + cloud formation → - air temperature	• + Large herbivores → - vegetation cover and density → - ET and - cloud cover → + air temperature<br>• + Large herbivores → - shrub and forest cover → + albedo and – atmospheric water vapor → - air temperature<br>• + Large herbivores → - shrub and forest cover → - snow masking → + snow cover duration → + albedo → - air temperature<br>• + Large herbivores → + BVOC release → + SOA → + albedo and cloud cover → - air temperature	• - Rodents → + field layer vegetation → - albedo → + air temperature<br>• - Rodents → + field layer vegetation → + snow masking → - snow cover duration → - albedo → + air temperature<br>• - Rodents → + field layer vegetation → + ET → + atmospheric water vapor → + air temperature<br>• - Rodents → + field layer vegetation → + ET → + latent heat absorbed and clouds→ - air temperature	• + Insects → - vegetation growth → - vegetation cover and density → - ET → - latent heat absorbed and – clouds → + air temperature<br>• + Insects → - vegetation growth → - vegetation cover and density → + albedo → - air temperature<br>• + Insects → - vegetation growth → - vegetation cover and density → - atmospheric water vapor → - GH effect → - air temperature<br>• + Insects → - vegetation growth → + snow masking → + snow season duration → - air temperature<br>• + Insects → + BVOC release → + SOA → + albedo and cloud cover→ - air temperature

BVOC	Biogenic Volatile Organic Compound
ET	Evapotranspiration
GH	Greenhouse
SOA	Secondary organic aerosol

List of abbreviations used in Table S5.

Table S6. Extreme biotic events as drivers of ecosystem change

Ecosystem Component	**EXTREME BIOTIC EVENTS**	
	**Increasing insect outbreaks**	**Decreasing peaks in rodent population**
**Local Climate**	• + Insect outbreaks → - vegetation cover and density → - ET → - latent heat absorbed and - cloud cover → + air temperature<br>• + Insect outbreaks → - vegetation cover and density → - snow masking → + snow cover duration → - air temperature<br>• + Insect outbreaks → - vegetation cover and density → + albedo and - atmospheric water vapor → - air temperature	• - Rodent peaks → + field layer vegetation → - albedo → + air temperature<br>• - Rodent peaks → + field layer vegetation → + snow masking → - snow cover duration → + air temperature

ET	Evapotranspiration

List of abbreviations used in Table S6.

Table S7. Local human activities as drivers of ecosystem change

Ecosystem Component	**Local human Activities**			
	**Decreasing Herding**	**Decreasing black carbon deposition**	**Expanding infrastructures**	**Decreasing Fire wood out-take**
**Local Climate**	• - Herding → + vegetation cover and density → - albedo → + air temperature<br>• - Herding → + vegetation cover and density → + vegetation masking → - snow cover duration → + air temperature<br>• - Herding → + vegetation cover and density → + ET and + cloud cover → - air temperature	• - BC → + snow albedo → - air temperature<br>• - BC → + snow albedo → - snow melt → + snow cover duration → - air temperature<br>• - BC → + lake ice albedo → + lake-ice duration → - air temperature	No major effect known	• - Fire wood out-take → + forest cover and density - - albedo → + air temperature<br>• - Fire wood out-take → + vegetation cover and density → + ET and cloud cover → - air temperature

BC	Black carbon
ET	Evapotranspiration

List of abbreviations used in Table S7.

Ecosystem component: Permafrost

Table S8. *Atmospheric drivers.*

Ecosystem Component	**Atmospheric drivers**					
	**Increasing air temperature**	**Increasing rainfall**	**Increasing ET**	**Decreasing UV-B radiation**	**Increasing Cloud cover**	**Increasing atmospheric CO_2 concentrations**
**Permafrost**	• + Air temperature → + ground temperature → + permafrost thaw<br>• + Air temperature → - snow season duration → + ground temperature → + permafrost thaw<br>• + Air temperature → + vegetation → + snow interception and - albedo → + ground temperature → + permafrost thaw<br>• + Air temperature → - lake ice season duration → + air temperature→ + ground temperature → + permafrost thaw<br>• + Air temperature → taller vegetation → + vegetation masking → - snow cover duration → + air and ground temperature → + permafrost thaw<br>• + Air temperature → + ET → + atmospheric water vapor → + GH effect → + air temperature → + permafrost thaw<br>• + Air temperature → + ET and – snow cover duration → - soil moisture in summer → - soil thermal conductivity → - permafrost thaw<br>• + Air temperature→ + vegetation → + LAI→ - ground temperature → - permafrost thaw<br>• + Air temperature → + BVOC → + SOA → - air temperature → - ground temperature → - permafrost thaw<br>• + Air temperature → + ET → + latent heat absorbed → - air temperature → - ground temperature → - permafrost thaw	• + Rainfall → + soil moisture and GWF → + thermal conductivity through the AL → + permafrost thaw<br>• + Rainfall → + plant productivity → - albedo and + snow interception → + ground warming → + permafrost thaw<br>• + Rainfall → taller vegetation → + vegetation masking → - snow cover duration → + air and ground temperature → + permafrost thaw<br>• + Rainfall → + ET→ + atmospheric water vapor → + GH effect → + air temperature → + permafrost thaw<br>• + Rainfall → + ET → + latent heat absorbed and + cloud cover → - air temperature → - permafrost thaw<br>• + Rainfall → + vegetation productivity → + LAI → - ground temperature → - permafrost thaw<br>• + Rainfall → + plant productivity → + vegetation cover and density → + BVOC → + SOA → - air temperature → - permafrost thaw	• + ET → + GH effect → warming → + permafrost thaw<br>• + ET → + latent heat absorbed → - air temperature → - permafrost thaw<br>• + ET → + water vapor → + cloud formation → - air temperature → - permafrost thaw<br>• + ET → - soil moisture and GWF → - permafrost thaw.	• - UV-B radiation → + vegetation cover and density → - albedo → + air temperature → + permafrost thaw<br>• - UV-B radiation → + vegetation cover and density → + snow interception → + permafrost thaw<br>• - UV-B radiation → + photosynthesis → + LAI → - permafrost thaw	• + Cloud cover → - ET → + RD and GWF → + permafrost thaw<br>• + Cloud cover→ overall – air temperature → - permafrost thaw	• + Atmospheric CO_2 → + CO_2 fertilization → + plant productivity → + vegetation cover and density → - albedo and + snow depth → + permafrost thaw<br>• + Atmospheric CO2 → taller vegetation → + vegetation masking → - snow cover duration → + air and ground temperature → + permafrost thaw<br>• + Atmospheric CO2 → closure of stomatal pores → - ET → + air temperature → + permafrost thaw<br>• + Atmospheric CO_2 → + vegetation cover and density → + LAI → - ground temperature → - permafrost thaw<br>• + Atmospheric CO_2 → + vegetation cover and density → + BVOC → + SOA → - air temperature → permafrost thaw<br>• + Atmospheric CO_2 → + vegetation cover and density → + ET and + cloud formation → - air temperature → -permafrost thaw

AL	Active layer
BVOC	Biogenic Volatile Organic Compound
CO_2	Carbon dioxide
ET	Evapotranspiration
GH	Greenhouse
GWF	Ground water flow
LAI	Leaf area index
SOA	Secondary organic aerosol

UV-B	Ultraviolet B

List of abbreviations used in Table S8.

Table S9. *Cryospheric drivers*

Ecosystem Component	**Cryospheric drivers**			
	**Decreasing snow cover duration**	**Increasing snow depth**	**Decreasing lake ice duration**	**Decreasing glacier extent**
**Permafrost**	• - Snow cover duration → - albedo and - latent heat absorbed → + air temperature → + permafrost thaw<br>• - Snow cover duration → overall + vegetation cover and density → - albedo, + atmospheric water vapor and + snow interception → + air and ground temperature → + permafrost thaw<br>• - Snow cover duration → overall + vegetation cover and density → + ET and + LAI index → - air and - ground temperature → - permafrost thaw<br>• - Snow cover duration → + heat and particulate release → - stable boundary layer → + mid-level cloud formation → - air temperature → - permafrost thaw<br>• - Snow cover duration → - summer soil moisture → - soil thermal conductivity → - permafrost thaw.	• + Snow depth → + insulation → + ground temperature → + permafrost thaw<br>• + Snow depth → + vegetation cover and density → - albedo and + snow interception → + air and ground temperature → + permafrost thaw<br>• + Snow depth → + plant productivity → + vegetation cover and density → + LAI index → - ground temperature → - permafrost thaw<br>• + Snow depth → + SWE → + latent heat absorbed during snow melt → - air temperature → - permafrost thaw	• - Lake ice duration → - albedo and + water vapor → + air temperature → + permafrost thaw<br>• - Lake ice duration → + rainfall → + soil moisture → + soil thermal conductivity → + permafrost thaw<br>• - Lake ice duration → overall + air temperature → + plant productivity → - albedo and + snow depth → + permafrost thaw<br>• - Lake ice duration → overall + air temperature → + plant productivity → + LAI → - permafrost thaw<br>• - Lake ice duration → + ET and cloud cover → - air temperature → - permafrost thaw.	• - Glacier extent → - albedo → + air temperature → + permafrost thaw

LAI	Leaf area index
SWE	Snow water equivalent

List of abbreviations used in Table S9.

Table S10. *Hydrospheric drivers*

Ecosystem Component	**Hydrospheric drivers**			
	**Decreasing soil moisture**	**Increasing SWE**	**Increasing RD and GWF**	**Increasing lake/river DOC concentrations**
**Permafrost**	• - Soil moisture → - ET, and + cloud cover → + air temperature → + permafrost thaw<br>• - Soil moisture → - plant productivity → - vegetation cover and density → - BVOC → - SOA and cloud cover → + air temperature → + permafrost thaw<br>• - Soil moisture → - vegetation cover → - LAI → + permafrost thaw<br>• - Soil moisture → - AL thermal conductivity → - permafrost thaw<br>• - Soil moisture → - forest and shrub cover → + albedo and - snow depth → - air and - ground temperature → - permafrost thaw<br>• - Soil moisture → - plant productivity → taller vegetation → - vegetation masking → + snow cover duration → - air and ground temperature → - permafrost thaw	• + SWE → + vegetation cover and density → - albedo and + atmospheric water wapor → + air temperature → + permafrost thaw<br>• + SWE → + ground water content upon snow melt → + soil thermal conductivity in summer → + permafrost thaw<br>• + SWE → + vegetation cover and density → - albedo and + snow interception → + ground temperature → + permafrost thaw<br>• + SWE → → taller vegetation → + vegetation masking → - snow cover duration → + air and ground temperature → + permafrost thaw<br>• + SWE → + vegetation cover and density → + LAI index → - ground temperature → - permafrost thaw<br>• + SWE → + ET → + cloud cover and + latent heat absorbed → - air temperature → - permafrost thaw	• + RD and GWF → + AL thermal conductivity → + permafrost thaw.	No major local effects known.

AL	Active Layer
BVOC	Biogenic Volatile Organic Compound
ET	Evapotranspiration
GWF	Ground water flow
LAI	Leaf area index
RD	River discharge
SOA	Secondary organic aerosol
SWE	Snow water equivalent

List of abbreviations used in Table S10.

Table S11. *Extreme abiotic events as drivers of ecosystem change*

Ecosystem Components	**Extreme abiotic events**			
	**Increasing WWE**	**Increasing extreme rainfall events**	**Increasing Droughts**	**Increasing wild-fires**
**Permafrost**	• + WWE → + ROS and WSE → - SWE → - snow cover duration → - albedo → + air and ground temperature → + permafrost thaw<br>• + WWE → + ROS and WSE → + vegetation damage → - shrub cover and density → - LAI → + permafrost thaw<br>• + WWE → + ROS and WSE → + vegetation damage → - shrub cover and density → - snow interception → - ground temperature → - permafrost thaw<br>• + WWE → + ROS and WSE → - shrub cover and density → + albedo → - ground temperature → - permafrost thaw<br>• + WWE → + ROS and WSE → + snowpack density and - snow depth → - insulation effect → - winter ground temperature → - permafrost thaw<br>• + WWE → + ROS and WSE → - summer soil moisture → - soil thermal conductivity → - permafrost thaw	• + ERE → + RD → + soil surface erosion → + permafrost thaw<br>• + ERE → soil moisture, RD and GWF → + thermal conductivity in AL → + permafrost thaw	• + Droughts → + fire → + permafrost thaw<br>• + Droughts → - plant productivity → - LAI → + permafrost thaw<br>• + Droughts → - ET → + air temperature → + permafrost thaw<br>• + Droughts → - soil moisture, RD and GWF → - soil thermal conductivity → - permafrost thaw<br>• + Droughts → - ET → - atmospheric water vapor → - GH effect → - air temperature → - permafrost thaw<br>• + Droughts → - vegetation cover and density → - surface albedo → - air temperature → - permafrost thaw	• + Fire → - surface OL → + soil thermal conductivity → + permafrost thaw<br>• + Fire → - forest and shrub cover → - ET → - latent heat absorbed and – cloud cover → + air temperature → + permafrost thaw<br>• + Fire → - forest and shrub cover → - BVOC → - SOA → - albedo and - cloud cover → + air temperature → + permafrost thaw<br>• + Fire → - forest and shrub cover → + albedo, - atmospheric water vapor, and - LAI → - ground temperature → - permafrost thaw<br>• + Fire → - forest and shrub cover → - snow interception → - winter insulation → - permafrost thaw<br>• + Fire → - vegetation masking → + snow cover duration → - air and ground temperature → - permafrost thaw

AL	Active layer
BVOC	Biogenic Volatile Organic Compound
ET	Evapotranspiration
ERE	Extreme precipitation event
GH	Greenhouse
GWF	Ground water flow
LAI	Leaf area index
OL	Organic layer
RD	River discharge
ROS	Rain on snow
SOA	Secondary organic aerosol
SWE	Snow water equivalent
WSE	Winter snowmelt event
WWE	Winter warming event

List of abbreviations used in Table S11.

Table S12. *Biospheric drivers*

Ecosystem Component	**Biosphere**				
	**Increasing Plant Productivity**	**Increasing BVOC emissions**	**Increasing large herbivores**	**Decreasing rodents population**	**Increasing insects population**
**Permafrost**	• + Plant productivity → + shrub and forest cover → + snow interception and - sublimation → + snow depth → + ground temperature → + permafrost thaw<br>• + Plant productivity → + shrub and forest cover → - albedo and + atmospheric water vapor → + air temperature → + permafrost thaw<br>• + Plant productivity → + snow masking → - snow cover duration → + air and ground temperature → + permafrost thaw<br>• + Plant productivity → + vegetation cover and density → + ET → + latent heat absorbed and + cloud cover → - air temperature → - permafrost thaw<br>• + Plant productivity → + vegetation cover and density → + BVOC → + SOA → + albedo and + cloud cover → - air temperature → - permafrost thaw<br>• + Plant productivity → + shrub and forest cover → + LAI index → - ground temperature → - permafrost thaw	• + BVOC → + SOA → + albedo and + cloud cover → - air temperature → - vegetation cover and density → - LAI → + ground temperature → + permafrost thaw<br>• + BVOC → + SOA → + diffuse radiation → + LUE → + vegetation cover and density → → + snow interception and - albedo → + permafrost thaw<br>• + BVOC → + SOA → + albedo and + cloud cover → - air temperature → - permafrost thaw<br>• + BVOC → overall - air temperature, → - vegetation cover and density → - snow interception and + albedo → - permafrost thaw<br>• + BVOC → + SOA and + cloud cover → + diffuse radiation → + LUE → + vegetation cover and density → → + LAI → - permafrost thaw	• + Large herbivores → + grazing and browsing → -vegetation cover and density → - LAI → + summer ground temperature → + permafrost thaw<br>• + Large herbivores → + grazing and browsing → - vegetation cover → overall – air temperature → - permafrost thaw<br>• + Large herbivores → + grazing and browsing → - vegetation cover and density → - snow interception → - winter ground temperature → - permafrost thaw<br>• + Large herbivores → - shrub and forest cover → - snow masking → + snow cover duration → - ground temperature → - permafrost thaw<br>• + Large herbivores → + BVOC release → + SOA → + albedo and cloud cover → - air temperature → - permafrost thaw	• - Rodents → + field layer vegetation → + snow interception → + winter ground temperature → + permafrost thaw<br>• - Rodents → + field layer vegetation → - albedo and + atmospheric water vapor → + air temperature → + permafrost thaw<br>• - Rodents → + field layer vegetation → + snow masking → - snow cover duration → - + air and ground temperature → + permafrost thaw<br>• - Rodents → + field layer vegetation → + ET → + latent heat absorbed and clouds → - air temperature → - permafrost thaw<br>• - Rodents → + field layer vegetation → + LAI index → - summer ground temperature → - permafrost thaw	• + Insects → - vegetation cover and density → - leaf density → - LAI index → + ground temperature → + permafrost thaw<br>• + Insects → overall - air temperature → - permafrost thaw<br>• + Insects → - vegetation cover and density → + snow masking → + snow season duration → - ground temperature → - permafrost thaw<br>• + Insects → - vegetation cover and density → - snow interception → - ground temperature → - permafrost thaw<br>• + Insects → + BVOC release → + SOA → + albedo and cloud cover→ - air temperature → - permafrost thaw

BVOC	Biogenic Volatile Organic Compound
ET	Evapotranspiration
LAI	Leaf area index
LUE	Light use efficiency
SOA	Secondary organic aerosol

List of abbreviations used in Table S12.

Table S13. *Extreme biotic events as drivers of ecosystem change*

Ecosystem Component	**EXTREME BIOTIC EVENTS**	
	**Increasing insect outbreaks**	**Decreasing peaks in rodent population**
**Permafrost**	• + Insect outbreaks → - vegetation cover and density → - LAI index → + ground temperature → + permafrost thaw<br>• + Insect outbreaks → - vegetation cover and density → - ET → - latent heat absorbed and - cloud cover → + air temperature → + permafrost thaw<br>• + Insect outbreaks → - vegetation cover and density → - snow interception → - snow depth → - winter ground temperature → -permafrost thaw<br>• + Insect outbreaks → - vegetation cover and density → + albedo and - atmospheric water vapor → - air temperature → - permafrost thaw<br>• + Insect outbreaks → - vegetation cover and density → - snow masking → + snow cover duration → - air and ground temperature → - permafrost thaw	• - Rodent peaks → + field layer vegetation → + snow depth → + winter ground temperature → + permafrost thaw<br>• - Rodent peaks → + field layer vegetation → - albedo → + air and ground temperature → + permafrost thaw<br>• - Rodent peaks → + field layer vegetation → + snow masking → - snow cover duration → + air and ground temperature<br>• - Rodent peaks → + field layer vegetation → + LAI index → +- ground temperature → - permafrost thaw

ET	Evapotranspiration
LAI	Leaf area index

List of abbreviations used in Table S13.

Table S14. *Local human activities as drivers of ecosystem change*

Ecosystem Component	**Local human Activities**			
	**Decreasing Herding**	**Decreasing black carbon deposition**	**Expanding infrastructures**	**Decreasing Fire wood out-take**
**Permafrost**	• - Herding → + vegetation cover and density → overall + air temperature → + permafrost thaw<br>• - Herding → + vegetation cover and density → + snow depth → + ground temperature → + permafrost thaw<br>• - Herding → + vegetation cover and density → + vegetation masking → - snow cover duration → + air and ground temperature → + permafrost thaw<br>• - Herding → + vegetation cover and density → + LAI index → - ground temperature → - permafrost thaw	• - BC → - air temperature → - ET → + soil moisture → + soil thermal conductivity → + permafrost thaw<br>• - BC → - snow melt → + snow season duration → + soil moisture, RD and GWF in late spring and summer → + soil thermal conductivity → + permafrost thaw<br>• - BC → + albedo and + snow cover duration → - air and ground temperature → - permafrost thaw	• + Infrastructures → altered soil thermal properties → + permafrost thaw	• - Fire wood out-take → + forest cover and density → - albedo and + snow depth → + air and ground temperature → + permafrost thaw<br>• - fire wood out-take → + forest cover and density → + LAI → - soil temperature → - permafrost thaw

BC	Black carbon
ET	Evapotranspiration
GWF	Ground water flow
LAI	Leaf area index
RD	River discharge

List of abbreviations used in Table S14.

Ecosystem component: Hydrology

Table S15. *Atmospheric drivers.*

Ecosystem Component	Atmospheric drivers					
	**Increasing air temperature**	**Increasing rainfall**	**Increasing ET**	**Decreasing UV-B radiation**	**Increasing Cloud cover**	**Increasing atmospheric CO_2 concentrations**
**Hydrology**	• + Air temperature → + BVOC → + SOA → + clouds → - ET → + RD and GWF<br>• + Air temperature → + permafrost thaw → + subsidence (mostly in lowlands) → + soil moisture<br>• + Air temperature → + ET → - RD and ground water flow<br>• + Air temperature → + permafrost thaw → + drainage (mostly in highlands) → - soil moisture<br>• + Air temperature → + vegetation cover and density → change in soil moisture	• + Rainfall → + RD and GWF<br>• + Rainfall → + soil moisture<br>• + Rainfall → overall + permafrost thaw → + subsidence (mostly in lowlands) → + soil moisture<br>• + Rainfall → overall + permafrost thaw → + drainage (mostly in highlands) → - soil moisture and – RD<br>• + Rainfall → + vegetation cover and density → change in soil moisture	• + ET → - soil moisture, - RD and - GWF	• - UV-B radiation → + plant productivity → overall + permafrost thaw → + subsidence (mostly in lowlands) → + soil moisture<br>• - UV-B radiation → → + plant productivity → overall + permafrost thaw → + drainage (mostly in highlands) → - soil moisture and – RD<br>• - UV-B radiation → + vegetation cover and density → change in soil moisture	• + Cloud cover→ overall – air temperature → - ET → + soil moisture, RD and GWF.	• + Atmospheric CO_2 → overall + permafrost thaw → + subsidence (mostly in lowlands) → + soil moisture<br>• + Atmospheric CO_2 → + vegetation cover and density → - RD during rain events<br>• + Atmospheric CO_2 → overall + permafrost thaw → + drainage (mostly in highlands) → - soil moisture and – RD<br>• + Atmospheric CO_2 → + vegetation cover and density → change in soil moisture

BVOC	Biogenic Volatile Organic Compound
CO_2	Carbon dioxide
ET	Evapotranspiration
GH	Greenhouse
GWF	Ground water flow
RD	River discharge
SOA	Secondary organic aerosol
UV-B	Ultraviolet B

List of abbreviations used in Table S15.

Table S16. *Cryospheric drivers*

Ecosystem Component	**Cryospheric drivers**				
	**Decreasing snow cover duration**	**Increasing snow depth**	**Increasing AL thickness**	**Decreasing lake ice duration**	**Decreasing glacier extent**
**Hydrology**	• - Snow cover duration → overall + permafrost thaw → + subsidence → + soil moisture<br>• - Snow cover duration → - water supply in spring and summer = - soil moisture, RD and GWF<br>• - Snow cover duration → overall + permafrost thaw → + drainage → - soil moisture<br>• - Snow cover duration → + vegetation cover and density → - RD in rain events<br>• - Snow cover duration → + vegetation cover and density → change in soil moisture	• + Snow depth → + SWE → + soil moisture, RD and GWF<br>• + Snow depth → overall + permafrost thaw → + subsidence → + soil moisture<br>• + Snow depth → overall + permafrost thaw → + drainage → - soil moisture and RD<br>• + Snow depth → + vegetation cover and density = - RD in rain events<br>• + Snow depth → + vegetation cover and density → change in soil moisture	• + AL thickness → + subsidence → + soil moisture<br>• + AL thickness → + drainage → - soil moisture and RD.	• - Lake ice duration → + evaporation and + cloud cover → - air temperature → less ET → + soil moisture, + RD, and + GWF<br>• - Lake ice duration → + evaporation on the lake → + water vapor → + precipitation → + soil moisture, RD and GWF<br>• - Lake ice duration → - albedo and + water vapor → + air temperature → + ET → - soil moisture, RD and GWF	• - Glacier extent → - water stored as ice → - ice melting → less RD and GWF.

AL	Active layer
ET	Evapotranspiration
RD	River discharge
GWF	Ground water flow

List of abbreviations used in Table S16.

Table S17. *Hydrospheric drivers*

Ecosystem Component	**Hydrospheric drivers**	
	**Decreasing soil moisture**	**Increasing SWE**
**Hydrology**	• - Soil moisture → overall - permafrost thaw → - drainage (mostly in highlands) → + soil moisture and RD<br>• - Soil moisture → - RD and GWF<br>• - Soil moisture → - wetland areas directly, and indirectly through - permafrost thaw and subsidence (mostly in lowlands)<br>• - Soil moisture → - vegetation cover → + RD during rain events	• + SWE → + soil surface moisture, RD and GWF<br>• + SWE → overall + permafrost thaw → + subsidence (mostly in lowlands) → + soil moisture<br>• + SWE → overall + permafrost thaw → + drainage (mostly in highlands) → - soil moisture<br>• + SWE → + vegetation cover and density → - RD during heavy rain events

GWF	Ground water flow
RD	River discharge
SWE	Snow water equivalent

List of abbreviations used in Table S17.

Table S18. *Extreme abiotic events as drivers of ecosystem change*

Ecosystem Components	**Extreme abiotic events**			
	**Increasing WWE**	**Increasing ERE**	**Increasing Droughts**	**Increasing wild-fires**
**Hydrology**	• + WWE → + ROS and WSE → + winter RD<br>• + WWE → + ROS and WSE → overall + permafrost thaw → + subsidence (mostly in lowlands) → + soil moisture<br>• + WWE → + ROS and WSE → - SWE → - late spring and summer soil moisture, RD and GWF<br>• + WWE → + ROS and WSE → overall + permafrost thaw → + drainage (mostly in highlands) → - soil moisture and RD	• + ERE → + soil moisture, RD and GWF.	• + Droughts → - soil moisture, RD and GWF<br>• + Droughts → - soil thermal conductivity → - permafrost thaw<br>• + Droughts → changes in permafrost thaw → changes in soil moisture, RD and GWF.	• + Fire → - vegetation cover and density → + RD during rain events<br>• + Fire → overall + permafrost thaw → + subsidence (mostly in lowlands) → + soil moisture<br>• + Fire → overall + permafrost thaw → + drainage (mostly in highlands) → - soil moisture and - RD<br>• + Fire → - vegetation cover and density → change in soil moisture

ERE	Extreme rainfall event
GWF	Ground water flow
RD	River discharge
ROS	Rain on snow
SWE	Snow water equivalent
WSE	Winter snowmelt event
WWE	Winter warming event

List of abbreviations used in Table S18.

Table S19. *Biospheric drivers*

Ecosystem Component	Biosphere				
	**Increasing Plant Productivity**	**Increasing BVOC emissions**	**Increasing large herbivores**	**Decreasing rodents population**	**Increasing insects population**
**Hydrology**	• + Plant productivity → overall + permafrost thaw → + subsidence (mostly in lowlands) → + soil moisture<br>• + Plant productivity → + vegetation cover and density → - RD during rain events<br>• + Plant productivity → overall + permafrost thaw → + drainage (mostly in highlands) → - soil moisture<br>• + Plant productivity → + vegetation cover and density → change in soil moisture	• + BVOC → - air temperature → - ET → + soil moisture, + RD and + GWF	• + Large herbivores → - vegetation cover → + RD during rain events<br>• + Large herbivores → - shrub and forest cover → - snow masking → + snow cover duration → + growing season soil moisture, RD and GWF<br>• + Large herbivores → - vegetation cover and density → change in soil moisture	• - Rodents → overall + permafrost thaw → + subsidence (mostly in lowlands) → + soil moisture<br>• - Rodents → + field layer vegetation → - RD during rain events<br>• - Rodents → overall + permafrost thaw → + drainage (mostly in highlands) → - soil moisture and – RD<br>• - Rodents → + field layer vegetation → → change in soil moisture	• + Insects → - vegetation cover and density → - changes in soil moisture

BVOC	Biogenic Volatile Organic Compound
ET	Evapotranspiration
GWF	Ground water flow
RD	River discharge

List of abbreviations used in Table S19.

Table S20. Extreme biotic events as drivers of ecosystem change

Ecosystem Component	**EXTREME BIOTIC EVENTS**	
	**Increasing insect outbreaks**	**Decreasing peaks in rodent population**
**Hydrology**	• + Insect outbreaks → - vegetation cover and density → + RD during heavy rainfall events<br>• + Insect outbreaks → - vegetation cover and density → changes in soil moisture	• - Rodent peaks → + field layer vegetation → overall + permafrost thaw → + subsidence (mostly in lowlands) → + soil moisture<br>• - Rodent peaks → + field layer vegetation → overall + permafrost thaw → + drainage (mostly in highlands) → - soil moisture and RD<br>• - Rodent peaks → + field layer vegetation → - RD during heavy rainfall events

RD	River discharge

List of abbreviations used in Table S20.

Table S21. *Local human activities as drivers of ecosystem change*

Ecosystem Component	**Local human Activities**			
	**Decreasing Herding**	**Decreasing black carbon deposition**	**Expanding infrastructures**	**Decreasing Fire wood out-take**
**Hydrology**	• - Herding → + vegetation cover and density → overall + permafrost thaw → + subsidence (mostly in lowlands) → + soil moisture<br>• - Herding → + vegetation cover and density → - RD during heavy rainfall events<br>• - Herding → + vegetation cover and density → overall + permafrost thaw → + drainage (mostly in highlands) → - soil moisture and – RD<br>• - Herding → + vegetation cover and density → changes in soil moisture	• - BC → - snow melt → + snow season duration → + soil moisture, RD and GWF in late spring and summer<br>• - BC → - air temperature → - ET → + soil moisture, RD and GWF	• + Infrastructures → altered soil thermal properties → + permafrost thaw → + subsidence (mostly in lowlands) → + soil moisture<br>• + Infrastructures → altered soil thermal properties → + permafrost thaw → + drainage (mostly in highlands) → - soil moisture and RD<br>• + Infrastructures → changes in water drainage and surface topography → local flooding, or drainage of lakes and ponds	• - Fire wood out-take → overall + permafrost thaw → + subsidence (mostly in lowlands) → + soil moisture<br>• - Fire wood out-take → + forest cover and density → - RD during heavy rainfall events<br>• - Fire wood out-take → overall + permafrost thaw → + drainage (mostly in highlands) → - soil moisture and - RD

BC	Black carbon
ET	Evapotranspiration
GWF	Ground water flow
RD	River discharge

List of abbreviations used in Table S21.

Ecosystem component: Vegetation

Table S22. *Atmospheric drivers.*

Ecosystem Component	**Atmospheric drivers**					
	**Increasing air temperature**	**Increasing rainfall**	**Increasing ET**	**Decreasing UV-B radiation**	**Increasing Cloud cover**	**Increasing atmospheric CO_2 concentrations**
**Vegetation**	• + Air temperature → + plant productivity<br>• + Air temperature → + permafrost thaw → + nutrient availability → + plant productivity<br>• + Air temperature → - lake ice season duration and + lake water temperature → + aquatic plant productivity<br>• + Air temperature → + BVOC release and + ET → + SOA and + clouds → + diffuse radiation → + (LUE) → + plant productivity<br>• + Air temperature → overall + permafrost thaw → + drainage (mostly in highlands) → - soil moisture → - plant productivity<br>• + Air temperature→ + ET → + water stress and + fires → - plant productivity<br>• + Air temperature → + insect herbivory → + vegetation damage → - plant productivity<br>• + Air temperature → + BVOC release and + ET → + SOA and + clouds → - air temperature → - plant productivity<br>• + Air temperature → + snow melt → early cut in water supply → + water stress → - plant productivity<br>• + Air temperature → + permafrost thaw and plant biomass → + lateral TOC transport → + brownification → - aquatic plant productivity<br>• + Air temperature → overall + permafrost thaw → + subsidence (mostly in lowlands) → + soil moisture → + wetland vegetation and – dry hummock vegetation	• + Rainfall → + soil moisture → + plant productivity<br>• + Rainfall → + ET → + atmospheric water vapor → + air temperature → + plant productivity<br>• + Rainfall → - fire occurrence → - plant productivity<br>• + Rainfall → overall + permafrost thaw → + nutrient availability → + plant productivity<br>• + Rainfall → + ET and + BVOC → + SOA and + cloud cover → + LUE → + plant productivity<br>• + Rainfall → + ET → + SOA and + cloud cover → - air temperature → - plant productivity<br>• + Rainfall → overall + permafrost thaw → + drainage (mostly in highlands) → - soil moisture → - plant productivity<br>• + Rainfall → + TOC transport = + brownification → - aquatic plant productivity<br>• + Rainfall → + soil moisture → + wetland vegetation<br>• + Rainfall → overall + permafrost thaw → + subsidence (mostly in lowlands) → + soil moisture → + wetland vegetation and – dry hummock vegetation<br>• + Rainfall → overall + permafrost thaw → + lake/pond drainage (mostly in highlands) → + dry hummock vegetation and - wetland vegetation	• + ET → + atmospheric water vapor → + GH effect → + air temperature → + plant productivity<br>• + ET → + cloud cover → + LUE → + plant productivity<br>• + ET → - soil moisture → + water stress → - plant productivity<br>• + ET → + water stress → + fire occurrence → - plant productivity<br>• + ET → + latent heat absorbed and + cloud cover → - air temperature → - plant productivity	• - UV-B radiation → + photosynthesis → + plant productivity<br>• - UV-B radiation → overall + permafrost thaw → + nutrient availability → + plant productivity<br>• - UV-B radiation → overall + permafrost thaw → + drainage (mostly in highlands) → - soil moisture → - plant productivity<br>• - UV-B radiation → overall + permafrost thaw → + subsidence (mostly in lowlands) → + soil moisture → + wetland vegetation and – dry hummock vegetation<br>• - UV-B radiation → overall + permafrost thaw → + drainage (mostly in highlands) → - soil moisture → + dry hummock vegetation and - wetland vegetation	• + Cloud cover → - ET → - water stress → + plant productivity<br>• + Cloud cover → + diffuse radiatrion → + LUE → + plant productivity<br>• + Cloud cover → overall – air temperature → - plant productivity	• + Atmospheric CO_2 → + CO_2 fertilization → + plant productivity<br>• + Atmospheric CO_2 → overall + permafrost thaw → + nutrient availability → + plant productivity<br>• + Atmospheric CO_2 → overall + permafrost thaw → + drainage (mostly in highlands) → - soil moisture → - plant productivity<br>• + Atmospheric CO_2 → overall + permafrost thaw → + subsidence (mostly in lowlands) → + soil moisture → + wetland vegetation and – dry hummock vegetation<br>• + Atmospheric CO_2 → overall + permafrost thaw → + lake/pond drainage (mostly in highlands) → + dry hummock vegetation and - wetland vegetation

	• + Air temperature → overall + permafrost thaw → + lake/pond drainage (mostly in highlands) → + dry hummock vegetation and - wetland vegetation					

BVOC	Biogenic Volatile Organic Compound
CO_2	Carbon dioxide
ET	Evapotranspiration
GH	Greenhouse
LUE	Light use efficiency
TOC	Total organic carbon
SOA	Secondary organic aerosol
UV-B	Ultraviolet B

List of abbreviations used in Table S22.

Table S23. *Cryospheric drivers*

Ecosystem Component	Cryospheric drivers				
	**Decreasing snow cover duration**	**Increasing snow depth**	**Increasing AL thickness**	**Decreasing lake ice duration**	**Decreasing glacier extent**
**Vegetation**	• - Snow cover duration → overall + air and ground temperature → + growing season → + plant productivity<br>• - Snow cover duration → overall + permafrost thaw → + nutrient availability → + plant productivity<br>• - Snow cover duration → + drought during growing season → - plant productivity<br>• - Snow cover duration → overall + permafrost thaw → + drainage (mostly in highlands) → - soil moisture → - plant productivity<br>• - Snow cover duration → overall + permafrost thaw → + subsidence (mostly in lowlands) → + wetland vegetation and – dry hummock vegetation<br>• - Snow cover duration → overall + permafrost thaw → + lake/pond drainage (mostly in highlands) → + dry hummock vegetation and – wetland vegetation<br>• - Snow cover duration → - snowbed vegetation communities	• + Snow depth → overall + ground temperature → + plant productivity<br>• + Snow depth → + SWE → + soil moisture → + plant productivity<br>• + Snow depth → overall + permafrost thaw → + nutrient availability → + plant productivity<br>• + Snow depth → + SWE → + latent heat absorbed during snowmelt → - air temperature → - plant productivity<br>• + Snow depth → overall + permafrost thaw → + drainage (mostly in highlands) → - soil moisture → - plant productivity<br>• + Snow depth → + avalanches → + vegetation damage → - plant productivity<br>• + Snow depth → + fungal infection → - plant productivity<br>• + Snow depth → overall + permafrost thaw → + subsidence (mostly in lowlands) → + soil moisture → + wetland vegetation and – dry hummock vegetation<br>• + Snow depth → overall + permafrost thaw → + lake/pond drainage (mostly in highlands) → + dry hummock vegetation and – wetland vegetation	• + AL thickness → + nutrient availability → + plant productivity<br>• + AL thickness → + drainage {mostly in highlands} → - soil moisture → - plant productivity<br>• + AL thickness → + surface instability → + slope processes → + vegetation damage → - plant productivity<br>• + AL thickness → + lake/pond drainage {mostly in highlands} → + dry hummock vegetation but - wetland vegetation<br>• + AL thickness → + subsidence {mostly in lowlands} → + soil moisture → + wetland vegetation but – dry hummock vegetation	• - Lake ice duration → + aquatic plant productivity<br>• - Lake ice duration → - albedo and + atm water vapor → + air temperature → + plant productivity<br>• - Lake ice duration → + precipitation → + plant productivity<br>• - Lake ice duration → overall + permafrost thaw → + nutrient availability → + plant productivity<br>• - Lake ice duration → + evaporation and + cloud cover → + LUE → + plant productivity<br>• - Lake ice duration → overall + air temperature → + permafrost thaw → + drainage (mostly in highlands) → - soil moisture → - plant productivity<br>• - Lake ice duration → + evaporation and + cloud cover → - air temperature → - plant productivity<br>• - Lake ice duration → overall + permafrost thaw → + soil moisture (mostly in lowlands) → + wetland vegetation and - dry hummock vegetation<br>• - Lake ice duration → overall + permafrost thaw → + lake/pond drainage (mostly in highlands) → - wetland vegetation and more dry hummock vegetation	• - Glacier extent → + deglaciated area → + vegetation expansion → + plant productivity<br>• - Glacier extent → + air temperature → + plant productivity<br>• - Glacier extent → - RD and GWF → - wetland areas → - wetland vegetation and + dry hummock vegetation

AL	Active layer
ET	Evapotranspiration
GWF	Ground water flow
LUE	Light use efficiency
RD	River discharge
SWE	Snow water equivalent

List of abbreviations used in Table S23.

__Table S24.__ Hydrospheric drivers

Ecosystem Component	**Hydrospheric drivers**			
	**Decreasing soil moisture**	**Increasing SWE**	**Increasing RD and GWF**	**Increasing lake/river DOC concentrations**
**Vegetation**	• - Soil moisture ⟶ overall - permafrost thaw ⟶ - drainage (mostly in highlands) ⟶ + soil moisture ⟶ + plant productivity<br>• - Soil moisture ⟶ - ET and - cloud cover ⟶ + air temperature ⟶ + plant productivity<br>• - Soil moisture ⟶ - plant productivity<br>• - Soil moisture ⟶ - vegetation cover and density ⟶ overall - air temperature ⟶ - plant productivity<br>• - Soil moisture ⟶ - AL thermal conductivity ⟶ summer ground warming ⟶ - plant productivity<br>• - Soil moisture ⟶ overall - permafrost thaw ⟶ - nutrient availability ⟶ - plant productivity<br>• - Soil moisture ⟶ - ET ⟶ - cloud cover ⟶ - LUE ⟶ - plant productivity<br>• + Soil moisture ⟶ + wetland areas ⟶ + wetland vegetation and – dry hummock vegetation<br>• + Soil moisture ⟶ overall + permafrost thaw ⟶ + subsidence (mostly in lowlands) ⟶ + soil moisture ⟶ + wetland vegetation and – dry hummock vegetation<br>• + Soil moisture ⟶ overall + permafrost thaw ⟶ + lake/pond drainage (mostly in highlands) ⟶ - soil moisture ⟶ + dry hummock vegetation and - wetland vegetation	• + SWE ⟶ + insulation ⟶ + ground temperature ⟶ + plant productivity<br>• + SWE ⟶ + soil moisture ⟶ + plant productivity<br>• + SWE ⟶ overall + permafrost thaw ⟶ + nutrient availability ⟶ + plant productivity<br>• + SWE ⟶ + soil moisture ⟶ + ET ⟶ + cloud cover ⟶ + LUE ⟶ + plant productivity<br>• + SWE ⟶ + ET and + snowmelt ⟶ - air temperature ⟶ - plant productivity<br>• + SWE ⟶ + ET ⟶ + cloud cover ⟶ - air temperature ⟶ - plant productivity<br>• + SWE ⟶ + soil moisture ⟶ + wetland vegetation and – dry hummock vegetation<br>• + SWE ⟶ + ground temperature ⟶ overall + permafrost thaw ⟶ + subsidence (mostly in lowlands) ⟶ + soil moisture ⟶ + wetland vegetation<br>• + SWE ⟶ + ground temperature ⟶ overall + permafrost thaw ⟶ + lake/pond drainage (mostly in highlands) ⟶ - soil moisture ⟶ - wetland vegetation and + dry hummock vegetation	• + RD and GWF ⟶ overall + permafrost thaw ⟶ + nutrient availability ⟶ + plant productivity<br>• + RD ⟶ + erosion ⟶ + damage to riverine vegetation ⟶ - plant productivity<br>• + RD and GWF ⟶ + permafrost thaw ⟶ + drainage (mostly in highlands) ⟶ - soil moisture ⟶ - plant productivity<br>• + RD and GWF ⟶ + permafrost thaw ⟶ + subsidence (mostly in lowlands) ⟶ + soil moisture ⟶ + wetland vegetation and – dry hummock vegetation<br>• + RD and GWF ⟶ + permafrost thaw ⟶ + lake/pond drainage (mostly in highlands) ⟶ - soil moisture ⟶ + dry hummock vegetation and – wetland vegetation	• + DOC concentration ⟶ + brownification ⟶ - solar radiation penetration ⟶ - aquatic plant productivity

AL	Active Layer
DOC	Dissolved organic carbon
ET	Evapotranspiration
GWF	Ground water flow
LUE	Light use efficiency
RD	River discharge
SWE	Snow water equivalent

List of abbreviations used in Table S24.

__Table S25.__ Extreme abiotic events as drivers of ecosystem change

Ecosystem Components	**Extreme abiotic events**			
	**Increasing WWE**	**Increasing ERE**	**Increasing Droughts**	**Increasing wild-fires**
**Vegetation**	• + WWE → + ROS and WSE → + herbivory population crashes → - grazing and browsing → + plant productivity<br>• + WWE → + ROS and WSE → - snow cover duration → - albedo → + air temperature → + plant productivity<br>• + WWE → + ROS and WSE → overall + permafrost thaw → + nutrient availability → + plant productivity<br>• + WWE → + ROS and WSE → + vegetation frost damage → - plant productivity<br>• + WWE → + ROS and WSE → overall + permafrost thaw → + drainage (mostly in highlands) → - soil moisture → - plant productivity<br>• + WWE → + ROS and WSE → - late spring and summer soil moisture → - plant productivity<br>• + WWE → + ROS and WSE → + slush torrents → + vegetation damage → - plant productivity<br>• + WWE → + ROS and WSE → overall + permafrost thaw → + subsidence (mostly in lowlands) → + soil moisture → + wetland vegetation but - dry hummock vegetation<br>• + WWE → + ROS and WSE → overall + permafrost thaw → + drainage (mostly in highlands) → - soil moisture → - wetland vegetation but + dry hummock vegetation	• + ERE → - rodents population→ + field-layer vegetation cover and density → + plant productivity<br>• + ERE → → overall + permafrost thaw → + nutrient availability → + plant productivity<br>• + ERE → + permafrost thaw → + drainage (mostly in highlands) → - soil moisture → - plant productivity<br>• + ERE → + slope processes and soil surface erosion → + vegetation damage → - plant productivity<br>• + ERE → + soil moisture → + wetland areas → + increased wetland vegetation and - dry hummock vegetation<br>• + ERE → + permafrost thaw → + subsidence (mostly in lowlands) → + soil moisture → + wetland vegetation but – dry hummock vegetation<br>• + ERE → + permafrost thaw → + drainage (mostly in highlands) → - soil moisture → - wetland vegetation but + dry hummock vegetation	• + Droughts → - soil moisture → - plant productivity<br>• + Droughts → + fire → - plant productivity<br>• + Droughts → - soil moisture → - wetland areas	• + Fire → overall + permafrost thaw → + nutrient availability → + plant productivity<br>• + Fire → + vegetation damage → - vegetation cover and density<br>• + Fire → - vegetation cover and density → overall – air temperature → - plant productivity<br>• + Fire → overall + permafrost thaw → + drainage (mostly in highlands) → - soil moisture → - plant productivity<br>• + Fire → removal of former vegetation cover → reforestation → potential surface colonization by new plant species → change in plant species composition<br>• + Fire → overall + permafrost thaw → + subsidence (mostly in lowlands) → + soil moisture → + wetland vegetation and – dry hummock vegetation<br>• + Fire → overall + permafrost thaw → + drainage (mostly in highlands) → - soil moisture → - wetland vegetation and + dry hummock vegetation

ERE	Extreme rainfall event
ROS	Rain on snow
WSE	Winter snowmelt event
WWE	Winter warming event

List of abbreviations used in Table S25.

Table S26. *Biospheric drivers*

Ecosystem Component	**Biosphere**			
	**Increasing BVOC emissions**	**Increasing large herbivores**	**Decreasing rodents population**	**Increasing insects population**
**Vegetation**	• + BVOC → + SOA and + cloud cover → + diffuse radiation → + plant productivity<br>• + BVOC → + SOA and + cloud cover → - air temperature → - plant productivity	• + Large herbivores → + BVOC → + SOA and cloud cover → + LUE → + plant productivity<br>• + Large herbivores → + grazing and browsing → - vegetation cover and density → - plant productivity	• - Rodents → + field layer vegetation<br>• - Rodents → overall + permafrost thaw → + nutrient availability → + plant productivity<br>• - Rodents → overall + permafrost thaw → + subsidence (mostly in lowlands) → + soil moisture → + wetland vegetation and – dry hummock vegetation<br>• - Rodents → overall + permafrost thaw → + lake/pond drainage (mostly in highlands) → - soil moisture → + dry hummock vegetation and - wetland vegetation	• + Insects → + vegetation damage → - plant productivity<br>• + Insects → overall - air temperature → - plant productivity<br>• + Insects → + BVOC → + SOA and + cloud cover → + diffuse radiation → + LUE → + plant productivity

BVOC	Biogenic Volatile Organic Compound
ET	Evapotranspiration
LUE	Light use efficiency
SOA	Secondary organic aerosol

List of abbreviations used in Table S26.

__Table S27.__ Extreme biotic events as drivers of ecosystem change

Ecosystem Component	**EXTREME BIOTIC EVENTS**	
	**Increasing insect outbreaks**	**Decreasing peaks in rodent population**
**Vegetation**	• + Insect outbreaks → + plant dieback → - vegetation cover and density → - plant productivity<br>• + Insect outbreaks → - vegetation cover and density → overall – air temperature → - plant productivity	• - Rodent peaks → + field layer vegetation cover and density<br>• - Rodent peaks → + field layer vegetation → - albedo → + air temperature → + plant productivity<br>• - Rodent peaks → overall + permafrost thaw → + nutrient availability → + plant productivity<br>• - Rodent peaks → overall + permafrost thaw → + drainage (mostly in highlands) → - soil moisture → - plant productivity<br>• - Rodent peaks → overall + permafrost thaw → + subsidence (mostly in lowlands) → + soil moisture → + wetland vegetation and – dry hummock vegetation<br>• - Rodent peaks → overall + permafrost thaw → + lake/pond drainage (mostly in highlands) → - soil moisture → + dry hummock vegetation and - wetland vegetation

Table S28. *Local human activities as drivers of ecosystem change*

Ecosystem Component	**Local human Activities**			
	**Decreasing Herding**	**Decreasing black carbon deposition**	**Expanding infrastructures**	**Decreasing Fire wood out-take**
**Vegetation**	• - Herding → - grazing and browsing → + plant productivity<br>• - Herding → + vegetation cover and density → - albedo → + air temperature → + plant productivity<br>• - Herding → + vegetation cover and density → overall + permafrost thaw → + drainage (mostly in highlands) → - soil moisture → - plant productivity<br>• - Herding → + vegetation cover and density → overall + permafrost thaw → + lake/pond drainage (mostly in highlands) → - soil moisture → + dry hummock vegetation and – wetland vegetation<br>• - Herding → + vegetation cover and density → overall + permafrost thaw → + subsidence (mostly in lowlands) → + soil moisture → + wetland vegetation and - dry hummock vegetation	• - BC → + spring-summer soil moisture → + plant productivity<br>• - BC → + albedo and + snow cover duration → - air temperature → - growing season → - plant productivity	• + Infrastructures → + deforestation → - vegetation cover and density → - plant productivity<br>• + Infrastructures → altered grazing pressure → increased grazing pressure in certain areas → - vegetation cover and density → - plant productivity<br>• + Infrastructures → local drainage or flooding → local changes in species composition	• - Fire wood out-take → + forest cover and density → + plant productivity<br>• - Fire wood out-take → overall + permafrost thaw → + subsidence (mostly in lowlands) → + soil moisture → + plant productivity<br>• - Fire wood out-take → overall + permafrost thaw → + subsidence (mostly in lowlands) → + soil moisture → + wetland vegetation and – dry hummock vegetation<br>• - Fire wood out-take → overall + permafrost thaw → + lake/pond drainage (mostly in highlands) → - soil moisture → + dry hummock vegetation and – wetland vegetation

BC	Black carbon

List of abbreviations used in Table S28.

Ecosystem component: Carbon Cycle

Table S29. *Atmospheric drivers.*

Ecosystem Component	**Atmospheric drivers**					
	**Increasing air temperature**	**Increasing rainfall**	**Increasing ET**	**Decreasing UV-B radiation**	**Increasing Cloud cover**	**Increasing atmospheric CO_2 concentrations**
**Carbon Cycle**	• + Air temperature → + plant productivity → + C uptake → + NEE<br>• + Air temperature → + permafrost thaw → + nutrient availability → + plant productivity → + C uptake → + NEE<br>• + Air temperature → + vegetation cover and density → + LAI → - summer topsoil temperature → - summer topsoil respiration → + NEE<br>• + Air temperature → overall + permafrost thaw → + subsidence (mostly in lowlands) → + soil moisture → + wetlands → + net C uptake → + NEE (but + CH_4 released)<br>• + Air temperature → - lake ice season duration and + lake water temperature → + aquatic plant productivity → + C uptake → + NEE<br>• + Air temperature → + ET → - RD and GWF→ - lateral transport of TOC → - C release → + NEE<br>• + Air temperature → + BVOC and + ET → + SOA and + clouds → + LUE → + plant productivity → + C uptake → + NEE<br><br>• + Air temperature → + fires → + C release → - NEE<br>• + Air temperature → + ground temperature → + soil respiration → → + C release → - NEE<br>• + Air temperature → + insect population and outbreak frequency → - plant productivity → - C uptake → - NEE<br>• + Air temperature → + ET→ + drought during growing season → - C uptake → - NEE<br>• + Air temperature → - lake ice season duration and + lake water temperature → + CO^2 and CH_4 release → - NEE<br>• + Air temperature → + permafrost thaw → + soil respiration → + C release → - NEE<br>• + Air temperature → + C emitted as BVOC → - NEE	• + Rainfall → + soil moisture → + plant productivity → + C uptake → + NEE<br><br>• + Rainfall → + ET → + atmospheric water vapor → + air temperature → + plant productivity → + C uptake → + NEE<br><br>• + Rainfall → - fire occurrence → + vegetation cover and density → + C uptake→ + NEE<br><br>• + Rainfall → + soil moisture → + wetlands → + C uptake → + NEE (but + CH_4 released)<br><br>• + Rainfall → + ET and + BVOC → + SOA and + cloud cover → + LUE → + plant productivity → + C uptake → + NEE<br><br>• + Rainfall → + vegetation cover and density → + LAI → - summer topsoil temperature → - summer topsoil respiration → + NEE<br><br>• + Rainfall → overall + permafrost thaw → + subsidence (mostly in lowlands) → + soil moisture → + wetlands → + net C uptake → + NEE (but + CH_4 released)<br><br>• + Rainfall → overall + permafrost thaw → + nutrient availability → + plant productivity → + C uptake → + NEE<br><br>• + Rainfall → + permafrost thaw → + soil respiration → + C release → - NEE<br><br>• + Rainfall → + permafrost thaw → + drainage (mostly in highlands) → - soil moisture → - plant productivity → - C uptake → - NEE	• + ET → + atmospheric water vapor → + GH effect + air temperature → + plant productivity → + C uptake → + NEE<br><br>• + ET → - RD and GWF → - transport of TOC → - C release → + NEE<br><br>• + ET → + latent heat absorbed and + cloud formation → - air temperature → - permafrost thaw → - C release → + NEE<br><br>• + ET → - soil moisture and GWF → - permafrost thaw → - C release → + NEE<br><br>• + ET → + cloud cover → + LUE → + plant productivity → + C uptake → + NEE<br><br>• + ET → + GH effect → warming → + permafrost thaw → + soil respiration → + C release → - NEE<br><br>• + ET → + water stress → - plant productivity → - C uptake → - NEE<br><br>• + ET→ + drought → + fire occurrence → + C release → - NEE<br><br>• + ET → + latent heat absorbed → - air temperature → - plant productivity → - C uptake → - NEE<br><br>• + ET → - soil moisture → changes in microbial activity → changes in C release → changes in NEE	• - UV-B radiation → + photosynthesis → + C uptake → + NEE<br><br>• - UV-B radiation → overall + permafrost thaw → + nutrient availability → + plant productivity → + C uptake → + NEE<br><br>• - UV-B radiation → overall + permafrost thaw → + subsidence (mostly in lowlands) → + soil moisture → + wetland vegetation → + NEE<br><br>• - UV-B radiation → - photodegradation → - C release → + NEE<br><br>• - UV-B radiation → overall + permafrost thaw → + soil respiration → + C release → - NEE<br><br>• - UV-B radiation → overall + permafrost thaw → + drainage (mostly in highlands) → - soil moisture → - plant productivity → - NEE<br><br>• - UV-B radiation → overall + permafrost thaw → + drainage (mostly in highlands) → - soil moisture → + dry hummock vegetation and - wetland vegetation → - NEE	• + Cloud cover → + LUE → + plant productivity → + C uptake → + NEE<br><br>• + Cloud cover → - ET → - water stress → + plant productivity → + C uptake → + NEE<br><br>• + Cloud cover → - air temperature → - permafrost thaw → - soil respiration → - C release → + NEE<br><br>• + Cloud cover → overall - air temperature and – permafrost thaw → - soil respiration → - C release → + NEE<br><br>• + Cloud cover → - air and ground temperature → - soil respiration → - C release → + NEE<br><br>• + Cloud cover → - air temperature → + lake ice duration and - lake water temperature → - CO^2 and CH_4 release → + NEE<br><br>• + Cloud cover → - air temperature → + lake ice season duration and - lake water temperature → - aquatic plant productivity → - C uptake → - NEE<br><br>• + Cloud cover → - air temperature → - plant productivity → - C uptake → - NEE<br><br>• + Cloud cover → - ET → + RD and GWF → + lateral TOC transport → + C release → - NEE	• + Atmospheric CO_2 → + plant productivity → + C uptake → + NEE<br><br>• - Atmospheric CO_2 → overall + permafrost thaw → + nutrient availability → + plant productivity → + C uptake → + NEE<br><br>• + Atmospheric CO_2 → overall + permafrost thaw → + subsidence (mostly in lowlands) → + soil moisture → + wetland s→ + net C uptake → + NEE (but + CH_4 released)<br><br>• Atmospheric CO_2 → + vegetation cover and density → + LAI → - summer topsoil temperature → - summer topsoil respiration → + NEE<br><br>• + Atmospheric CO_2 → overall + permafrost thaw → + soil respiration → C release → - NEE<br><br>• + Atmospheric CO_2 → overall + permafrost thaw → + lake/pond drainage (mostly in highlands) → + soil respiration → + CO^2 release → - NEE (but – CH_4 released)<br><br>• + Atmospheric CO_2 → overall + permafrost thaw → + drainage (mostly in highlands) → - soil moisture → - plant productivity → - NEE<br><br>• + Atmospheric CO_2 → + soil respiration in autumn and winter → + C release → - NEE<br><br>• + Atmospheric CO_2 → + plant biomass and overall - permafrost thaw → + lateral TOC transport → + C release → - NEE

	• + Air temperature → + BVOC and + ET → + SOA and + clouds → - air temperature → - plant productivity<br>• + Air temperature → overall + permafrost thaw → + lake/pond drainage (mostly in highlands) → + soil respiration → + CO^2 release → - NEE (but – CH_4 released)<br>• + Air temperature → overall + permafrost thaw → + drainage (mostly in highlands) → - soil moisture → - plant productivity → - NEE<br>• + Air temperature → + plant biomass and + permafrost thaw → + lateral transport of TOC→ + C release → - NEE<br>• + Air temperature → overall + lateral transport of TOC → + lake brownification → - aquatic plant productivity → - C uptake → - NEE<br>• + Air temperature → + ET → - soil moisture → changes in soil respiration → changes in NEE<br>• + Air temperature → + vegetation cover and density → changes in soil moisture → changes in soil respiration → changes in NEE	• + Rainfall → + ET → + SOA and + cloud cover → - air temperature → - plant productivity → - C uptake → - NEE<br>• + Rainfall → overall + permafrost thaw → + lake/pond drainage (mostly in highlands) → + soil respiration → + CO^2 release → - NEE (but – CH_4 released)<br>• + Rainfall → overall + permafrost thaw, + plant biomass and + RD → + lateral TOC transport → + C release → - NEE<br>• + Rainfall → + permafrost thaw, + plant biomass and + RD → + lateral TOC transport → + brownification → - C uptake → - NEE<br>• + Rainfall → + soil moisture → changes in microbial activity → changes in soil respiration → changes in NEE<br>• + Rainfall → + vegetation cover and density → changes in soil moisture → changes in soil respiration → changes in NEE			• + Cloud cover → - ET → + soil moisture → changes in microbial activity → changes in C release → changes in NEE	• + Atmospheric CO_2 → + plant biomass and overall - permafrost thaw → + lateral TOC transport → + brownification → - aquatic plant productivity → - C uptake → - NEE<br>• + Atmospheric CO_2 → - BVOC flux per leaf , but far + biomass → + BVOC release → - NEE<br>• + Atmospheric CO_2 → + vegetation cover and density → change in soil moisture → changes in soil respiration → changes in NEE

BVOC	Biogenic Volatile Organic Compound
C	Carbon
CH_4	Methane
CO_2	Carbon dioxide
ET	Evapotranspiration
GH	Greenhouse
GWF	Ground water flow
LAI	Leaf area index
LUE	Light use efficiency
NEE	Net primary production
RD	River discharge
TOC	Total organic carbon
SOA	Secondary organic aerosol
UV-B	Ultraviolet B

List of abbreviations used in Table S29.

Table S30. *Cryospheric drivers*

Ecosystem Component	**Cryospheric drivers**				
	**Decreasing snow cover duration**	**Increasing snow depth**	**Increasing AL thickness**	**Decreasing lake ice duration**	**Decreasing glacier extent**
**Carbon Cycle**	• - Snow cover duration → overall + plant productivity → + C uptake → + NEE<br>• - Snow cover duration → overall + vegetation cover and density → + LAI → - summer topsoil temperature → - summer topsoil respiration → + NEE<br>• - Snow cover duration → overall + permafrost thaw → + nutrient availability → + plant productivity → + C uptake → + NEE<br>• - Snow cover duration → overall + permafrost thaw → + subsidence (mostly in lowlands)→ + wetland areas → + net C uptake → + NEE (but + CH_4 released)<br>• - Snow cover duration → + air temperature → - lake ice season duration and + lake water temperature → + aquatic plant productivity → + C uptake → + NEE<br>• - Snow cover duration → + drainage and – soil moisture → - RD → - lateral TOC transport → - C release → + NEE<br>• - Snow cover duration → + drought during growing season → - plant productivity → - C uptake → - NEE<br>• Snow cover duration → + ground temperature → + soil respiration → + C release → - NEE<br>• Snow cover duration → + air temperature → - lake ice season duration and + lake water temperature → + microbial activity → + CO^2 and CH_4 release → - NEE<br>• - Snow cover duration → overall + permafrost thaw → + soil respiration → + C release → - NEE<br>• - Snow cover duration → overall + permafrost thaw → + lake/pond drainage (mostly in highlands) → + soil respiration → + CO^2 release → - NEE (but – CH_4 released)	• + Snow depth → + growing season → + plant productivity → + C uptake → + NEE<br>• + Snow depth → overall + permafrost thaw → + nutrient availability → + plant productivity → + C uptake → + NEE<br>• + Snow depth → overall + vegetation cover and density → + LAI → - summer topsoil temperature → - summer topsoil respiration → + NEE<br>• + Snow depth → overall + permafrost thaw → + subsidence (mostly in lowlands) → + soil moisture → + wetland vegetation and – dry hummock vegetation → + NEE (but + CH_4 released)<br>• + Snow depth → + snow on lake-ice → - ice thickness → - lake-ice duration → + aquatic plant productivity → + NEE<br>• + Snow depth → + winter ground temperature → + winter soil respiration → + C release → - NEE<br>• + Snow depth → overall + permafrost thaw → + soil respiration → + C release → - NEE<br>• + Snow depth → → overall + permafrost thaw → + drainage (mostly in highlands) → - soil moisture → - plant productivity → - C uptake → - NEE<br>• + Snow depth → overall + permafrost thaw → + lake/pond drainage (mostly in highlands) → + soil respiration → + CO^2 release → - NEE (but – CH_4 released)<br>• + Snow depth → + fungal infection → - plant productivity → - NEE	• + AL thickness → nutrient availability → + plant productivity → + C uptake → + NEE<br>• + AL thickness → + subsidence (mostly in lowlands) → + soil moisture → + wetlands → + C uptake → + NEE (but + CH_4 released)<br>• + AL thickness → (mostly in highlands) + drainage → - plant productivity and - RD → - lateral TOC transport → - C release → + NEE<br>• + AL thickness → + exposure of labile C to remineralization → + C release → - NEE<br>• + AL thickness → {mostly in highlands} + drainage → - soil moisture → - plant productivity → - C uptake → - NEE<br>• + AL thickness → {mostly in highlands} + drainage → + soil respiration → + CO^2 release → - NEE (but – CH_4 released)<br>• + AL thickness → + C exposed to erosion → + lateral TOC transport → + C release → - NEE<br>• + AL thickness → (mostly in lowlands) + soil moisture → + plant productivity and + wetlands → + lateral TOC transport → + C release → - NEE<br>• + AL thickness → overall + lateral TOC transport → + brownification → - aquatic plant productivity → - C uptake → - NEE<br>• + AL thickness → + slope processes → - vegetation cover and density → - C uptake → - NEE	• - Lake ice duration → overall + air temperature → + plant productivity → + C uptake → + NEE<br>• - Lake ice duration → + rainfall → + plant productivity → + NEE<br>• - Lake ice duration → + rainfall → + soil moisture → + wetland areas → + NEE (but + CH_4 released)<br>• - Lake ice duration → + aquatic plant productivity → + C uptake → + NEE<br>• - Lake ice duration → overall + permafrost thaw → + nutrient availability → + plant productivity → + C uptake → + NEE<br>• - Lake ice duration → overall + permafrost thaw → + soil moisture (mostly in lowlands) → + wetland vegetation and - hummocky areas → + NEE (but + CH_4 released)<br>• - Lake ice duration → + evaporation and + cloud cover → + LUE → + plant productivity→ + C uptake → + NEE<br>• - Lake ice duration → + evaporation and + cloud cover → - air temperature → - plant productivity → - C uptake → - NEE<br>• - Lake ice duration → - albedo → + air temperature → + ground temperature → + soil respiration → + C release → - NEE<br>• - Lake ice duration → overall + permafrost thaw → + soil respiration → + C release → - NEE<br>• - Lake ice duration → overall + permafrost thaw → + lake/pond drainage (mostly in highlands) → + soil respiration → + CO^2 release → - NEE (but – CH_4 released)<br>• - Lake ice duration → overall + permafrost thaw → + drainage (mostly in highlands) → - soil moisture → - plant productivity	• - Glacier extent → + vegetation expansion → + C uptake → + NEE<br>• - glacier extent → - RD and GWF → - lateral TOC transport → - C release → + NEE<br>• - Glacier extent → - wetland areas → - net C uptake → - NEE

	• - Snow cover duration → overall + permafrost thaw → + drainage (mostly in highlands) → - soil moisture → - plant productivity → - NEE<br><br>• - Snow cover duration → + plant biomass, + permafrost thaw and + subsidence → + lateral TOC transport → + C release → - NEE<br><br>• - Snow cover duration → + plant biomass, + permafrost thaw and + subsidence → + lateral TOC transport → + brownification → less aquatic plant productivity → - C uptake → - NEE<br><br>• - Snow cover duration → - soil moisture → changes in microbial activity → changes in C release → changes in NEE<br><br>• - Snow cover duration → + vegetation cover and density → changes in soil moisture → changes in soil respiration → changes in NEE	• + Snow depth → + avalanches → + vegetation damage → - C uptake → - NEE<br><br>• + Snow depth → + snow on lake-ice → - ice thickness → - lake-ice duration → + water and sediment temperature → + microbial activity → + CO^2 and CH_4 release → - NEE<br><br>• + Snow depth → overall + plant biomass, + permafrost thaw and + RD during melt season → + lateral TOC transport → + C release → - NEE<br><br>• + Snow depth → overall + lateral TOC transport → lake brownification → - aquatic plant productivity → - NEE<br><br>• + Snow depth → + soil moisture → changes in microbial activity → changes in C release → changes in NEE	• + AL → changes in soil moisture → changes in microbial activity → changes in C release → changes in NEE	• - Lake ice duration → overall + air temperature → + soil respiration → - NEE<br><br>• - Lake ice duration → + water and sediment temperature → + CH_4 and CO^2 release → - NEE<br><br>• - Lake ice duration → overall + plant biomass, permafrost thaw and RD → + lateral TOC transport → + C release → - NEE<br><br>• - Lake ice duration → overall + plant biomass, permafrost thaw and RD → + lateral TOC transport → browinification → - aquatic plant productivity → - NEE<br><br>• - Lake ice duration → + rainfall → + soil moisture → changes in soil respiration → changes in NEE	

AL	Active layer
ET	Evapotranspiration
C	Carbon
CH_4	Methane
CO_2	Carbon dioxide
GWF	Ground water flow
LAI	Leaf area index
LUE	Light use efficiency
NEE	Net primary production
RD	River discharge
SWE	Snow water equivalent
TOC	Total organic carbon

List of abbreviations used in Table S30.

Table S31. *Hydrospheric drivers*

Ecosystem Component	**Hydrospheric drivers**			
	**Decreasing soil moisture**	**Increasing SWE**	**Increasing RD and GWF**	**Increasing lake/river DOC concentrations**
**Carbon Cycle**	• - Soil moisture → - ET and - cloud cover → + air temperature → + plant productivity → + C uptake → + NEE<br>• - Soil moisture → - permafrost thaw → - soil respiration → - C release → + NEE<br>• - Soil moisture → overall - permafrost thaw → - lake/pond drainage (mostly in highlands) → - soil respiration → - CO^2 release → + NEE (but + CH_4 released)<br>• - Soil moisture → overall - permafrost thaw → - drainage (mostly in highlands) → + soil moisture → + plant productivity → + NEE<br>• - Soil moisture → - plant biomass, - permafrost thaw, - wetland areas and - RD → - lateral TOC transport→ - C release → + NEE<br>• - Soil moisture → - lateral TOC transport → - brownification → + aquatic plant productivity → + NEE<br>• - Soil moisture → - plant productivity → - C uptake → - NEE<br>• - Soil moisture → - ET → - cloud cover → - LUE → - plant productivity → - C uptake → - NEE<br>• - Soil moisture → - vegetation cover and density → - LAI → + summer topsoil temperature → + summer topsoil respiration → - NEE<br>• - Soil moisture → - wetland areas → - net C uptake → - NEE (but - CH_4 released)<br>• - Soil moisture → overall - permafrost thaw → - nutrient availability → - plant productivity → - C uptake → - NEE<br>• - Soil moisture → overall - permafrost thaw → - subsidence (mostly in lowlands) → - soil moisture → - wetland vegetation → - NEE (but - CH_4 released)<br>• - Soil moisture → - vegetation cover → overall - air temperature → - plant productivity → - C uptake → - NEE<br>• - Soil moisture → changes in soil respiration → changes in NEE	• + SWE → + ground temperature and + soil moisture → + plant productivity → + C uptake → + NEE<br>• + SWE → + soil moisture → + ET → + cloud cover → + LUE → + C uptake → + NEE<br>• + SWE → + vegetation cover and density → + LAI → - summer summer topsoil temperature → - summer topsoil respiration → + NEE<br>• + SWE → overall + permafrost thaw → + nutrient availability → + plant productivity → + C uptake → + NEE<br>• + SWE → + soil moisture directly and through + permafrost thaw and subsidence (mostly in lowlands) → + wetland areas → + net C uptake → + NEE (but + CH_4 released)<br>• + SWE → overall + permafrost thaw → + soil respiration → + C release → - NEE<br>• + SWE → + snowmelt → - latent heat absorbed → - air temperature → - plant productivity → - C uptake → - NEE<br>• + SWE → + soil moisture → + ET → + latent heat absorbed and + cloud cover → - air temperature → - plant productivity → - C uptake → - NEE<br>• + SWE → overall + permafrost thaw → + lake/pond drainage (mostly in highlands) → + soil respiration → + CO^2 release → - NEE (but – CH_4 released)<br>• + SWE → overall + permafrost thaw → + drainage (mostly in highlands) → - soil moisture → - plant productivity → - NEE<br>• + SWE → + winter ground temperature → + winter soil respiration → + C release → - NEE<br>• + SWE → overall + plant biomass, + permafrost thaw and + RD → + lateral TOC transport → + C release → - NEE<br>• + SWE → overall + plant biomass, + permafrost thaw and + RD → + lateral TOC transport → + lake brownification → - aquatic plant productivity → - NEE<br>• + SWE → + soil moisture → → changes in soil respiration → changes in NEE	• + RD and GWF → + permafrost thaw → + subsidence (mostly in lowlands) → + soil moisture → + wetland vegetation and – dry hummock vegetation → + NEE (but + CH_4 released)<br>• + RD and GWF → overall + permafrost thaw → + nutrient availability → + plant productivity → + C uptake → + NEE<br>• + RD and GWF → + permafrost thaw → + soil respiration → + C release → - NEE<br>• + RD and GWF → + permafrost thaw → + drainage (mostly in highlands) → - soil moisture → - plant productivity → - NEE<br>• + RD and GWF → + permafrost thaw → + lake/pond drainage (mostly in highlands) → + soil respiration → + CO^2 release → - NEE (but – CH_4 released)<br>• + RD → + erosion → + damage to riverine vegetation → - plant productivity → - NEE<br>• + RD and GWF → overall increase in lateral TOC transport → + C release → - NEE<br>• + RD and GWF → overall increase in lateral TOC transport → + brownification → - aquatic plant productivity → - NEE	• + River-lake DOC concentration → + C release → - NEE<br>• + River-lake DOC concentration → + brownification → aquatic plant productivity → - C uptake → - NEE

AL	Active Layer
C	Carbon
CH_4	Methane
CO_2	Carbon dioxide
DOC	Dissolved organic carbon
ET	Evapotranspiration
GWF	Ground water flow
LAI	Leaf area index
LUE	Light use efficiency
NEE	Net primary production
RD	River discharge
SWE	Snow water equivalent
TOC	Total organic carbon

List of abbreviations used in Table S31.

Table S32. *Extreme abiotic events as drivers of ecosystem change*

Ecosystem Components	**Extreme abiotic events**			
	**Increasing WWE**	**Increasing ERE**	**Increasing Droughts**	**Increasing wild-fires**
**Carbon Cycle**	• + WWE → + ROS and WSE → + herbivory population crashes → - grazing and browsing → + plant productivity → + NEE<br>• + WWE → + ROS and WSE → - snow cover duration → - albedo → + air temperature → + plant productivity → + NEE<br>• + WWE → + ROS and WSE → + snow thermal conductivity and - snow depth → - winter ground temperature → - permafrost thaw and - winter soil respiration → - C release → + NEE<br>• + WWE → + ROS and WSE → overall + permafrost thaw → + nutrient availability → + plant productivity → + NEE<br>• + WWE → + ROS and WSE → overall + permafrost thaw → + subsidence (mostly in lowlands) → + soil moisture → + wetland vegetation → + NEE (but + CH_4 released)<br>• + WWE → + ROS and WSE → - snow on lake ice → - radiation interception → + aquatic plant productivity → + C uptake → + NEE<br>• + WWE → + ROS and WSE → overall - plant productivity → - lateral TOC transport → - C release → + NEE<br>• + WWE → + ROS and WSE → - spring and summer RD → - lateral TOC transport → - C release → + NEE<br>• + WWE → + ROS and WSE → overall + permafrost thaw → + soil respiration → + C release → - NEE<br>• + WWE → + ROS and WSE → overall + permafrost thaw → + drainage (mostly in highlands) → - soil moisture → - plant productivity → - NEE<br>• + WWE → + ROS and WSE → overall + permafrost thaw → + lake/pond drainage (mostly in highlands) + soil respiration → + CO^2 release → - NEE (but – CH_4 released)<br>• + WWE → + ROS and WSE → + vegetation frost damage → - plant productivity → - NEE<br>• + WWE → + ROS and WSE → - late spring and summer soil moisture → - plant productivity → - NEE<br>• + WWE → + ROS and WSE → + slush torrents → + vegetation damage → - plant productivity → - NEE	• + ERE → - rodents population → + field-layer vegetation cover and density → + C uptake → + NEE<br>• + ERE → + wetland areas → + net C uptake → + NEE<br>• + ERE → → overall + permafrost thaw → + nutrient availability → + plant productivity → + NEE<br>• + ERE → + permafrost thaw → + subsidence (mostly in lowlands) → + soil moisture → + wetland vegetation → + NEE (but + CH_4 released)<br>• + ERE → + permafrost thaw → + soil respiration → + C release → - NEE<br>• + ERE → + permafrost thaw → + drainage (mostly in highlands) → - soil moisture → - plant productivity + ERE → + permafrost thaw → + drainage (mostly in highlands) → - soil moisture → - NEE<br>• + ERE → + permafrost thaw → + lake/pond drainage (mostly in highlands) + soil respiration → + CO^2 release → - NEE (but – CH_4 released)<br>• + ERE → + slope processes and surface erosion → + vegetation damage → - C uptake → - NEE<br>• + ERE → + permafrost thaw, + wetlands and + RD → + lateral TOC transport → + C release → - NEE<br>• + ERE → + lateral TOC transport → + brownification → - aquatic plant productivity → - NEE	• + Droughts → - soil moisture → - soil respiration (not in wetlands) → + NEE<br>• + Droughts → - soil moisture in wetlands → + soil respiration → - NEE<br>• + Droughts → - cloud cover → - diffuse radiation → - LUE → - plant productivity → - NEE<br>• + Droughts → - soil moisture → - plant productivity → - C uptake → - NEE<br>• + Droughts → + fire → + C release → - NEE<br>• + Droughts → overall + permafrost thaw → + soil respiration → + C release → - NEE<br>• + Droughts → - wetland areas → - net C uptake → - NEE (but - CH_4 released)	• + Fire → - vegetation cover and density → - snow depth → - insulation → - winter ground temperature → - winter soil respiration → + NEE<br>• + Fire → overall + permafrost thaw → + nutrient availability → + plant productivity → + NEE<br>• + Fire → overall + permafrost thaw → + subsidence (mostly in lowlands) → + soil moisture → + wetland vegetation and – dry hummock vegetation → + NEE (but + CH_4 released)<br>• + Fire → - plant biomass → - lateral TOC transport → - C release → + NEE<br>• + Fire → - vegetation cover and density → - LAI → + summer topsoil temperature → + summer topsoil respiration → - NEE<br>• + Fire → + C release from burnt vegetation → - NEE<br>• + Fire → - vegetation cover and density → - C uptake → - NEE<br>• + Fire → - vegetation cover and density → overall – air temperature → - plant productivity → - C uptake → - NEE<br>• + Fire → overall + permafrost degradation → + soil respiration → + C release → - NEE<br>• + Fire → overall + permafrost thaw → + drainage (mostly in highlands) → - soil moisture → - plant productivity → - NEE<br>• + Fire → overall + permafrost thaw → + lake/pond drainage (mostly in highlands) + soil respiration → + CO^2 release → - NEE (but – CH_4 released)<br>• + Fire → removal of former vegetation cover → reforestation → potential surface colonization by new plant species → change in plant species composition → changes in net C uptake → changes in NEE<br>• + Fire → - vegetation cover and density → change in soil moisture → changes in soil respiration → changes in NEE

	• + WWE → + ROS and WSE → overall + permafrost thaw → + lateral TOC transport → + C release → - NEE<br>• + WWE → + ROS and WSE → + permafrost thaw → + lateral transport of TOC → + brownification → - aquatic plant productivity → - NEE<br>• + WWE → + ROS and WSE → - snow on lake-ice → - insulation → + ice thickness → + lake-ice season → - aquatic plant productivity → - NEE<br>• + WWE → + ROS and WSE → - late spring and summer soil moisture → changes in soil respiration → changes in NEE			

ERE	Extreme rainfall event
C	Carbon
CH_4	Methane
CO_2	Carbon dioxide
LAI	Leaf area index
LUE	Light use efficiency
NEE	Net primary production
RD	River discharge
ROS	Rain on snow
TOC	Total organic carbon
WSE	Winter snowmelt event
WWE	Winter warming event

List of abbreviations used in Table S32.

Table S33. *Biospheric drivers*

Ecosystem Component	**Biosphere**				
	**Increasing Plant Productivity**	**Increasing BVOC emissions**	**Increasing large herbivores**	**Decreasing rodents population**	**Increasing insects population**
**Carbon Cycle**	• + Plant productivity → + C uptake → + NEE<br>• + Plant productivity → + vegetation cover and density → + ET and + BVOC → + cloud cover → + LUE → + plant productivity → + C uptake → + NEE<br>• + Plant productivity → + vegetation cover and density → + LAI → - summer topsoil temperature → - topsoil respiration → + NEE<br>• + Plant productivity → overall + permafrost thaw → + nutrient availability → + plant productivity → + C uptake → + NEE<br>• + Plant productivity → overall + permafrost thaw → + subsidence (mostly in lowlands) → soil moisture → + wetland vegetation and - dry hummock vegetation → + NEE (but + CH_4 released)<br>• + Plant productivity → overall + air temperature and + winter ground temperature → + permafrost thaw and + winter soil respiration → + C release → - NEE<br>• + Plant productivity → + vegetation cover and density → + snow depth → + winter ground temperature → + winter soil respiration → - NEE<br>• + Plant productivity → overall + permafrost thaw → + drainage (mostly in highlands) → - soil moisture → - plant productivity → - NEE<br>• + Plant productivity → overall + permafrost thaw → + lake/pond drainage (mostly in highlands) + soil respiration → + CO^2 release → - NEE (but – CH_4 released)<br>• + Plant productivity → vegetation cover and density → + ET and + BVOC → - air temperature, → - plant productivity → - C uptake → - NEE	• + BVOC → overall – air temperature → - ground temperature → - soil respiration → + NEE<br>• + BVOC → + SOA → + cloud cover → + diffuse radiation → + plant productivity → + NEE<br>• + BVOC → + SOA → + cloud cover → - air temperature → - plant productivity → - NEE<br>• + BVOC → changes in plant productivity due to – air temperature and + diffuse radiation → small changes in ground temperature → small changes in permafrost thaw and soil respiration	• + Large herbivores → - vegetation cover and density → - snow depth → - winter ground temperature and overall - permafrost thaw → - soil respiration → - C release → + NEE<br>• + Large herbivores → + grazing and browsing → overall - plant biomass and overall - permafrost thaw → - lateral TOC transport → - C release → + NEE<br>• + Large herbivores → + BVOC → + SOA → + cloud cover → + LUE → + plant productivity → + C uptake → + NEE<br>• + Large herbivores → - vegetation cover and density → - LAI → + summer summer topsoil temperature → + summer topsoil respiration → - NEE<br>• + Large herbivores → + grazing and browsing → - vegetation cover and density → - plant productivity → - C uptake → - NEE<br>• + Large herbivores → + BVOC → + direct C release → → - NEE<br>• + Large herbivores → - vegetation cover and density → changes in soil moisture → changes in soil respiration → changes in NEE	• - Rodents → + field layer vegetation → + C uptake → + NEE<br>• - Rodents → + field layer vegetation → + LAI → - summer topsoil temperature → - topsoil respiration → + NEE<br>• - Rodents → overall + permafrost thaw → + nutrient availability → + plant productivity → + C uptake → + NEE<br>• - Rodents → overall + permafrost thaw → + subsidence (mostly in lowlands) → + wetland vegetation and – dry hummock vegetation → + net C uptake → + NEE (but + CH_4 released)<br>• - Rodents → + field layer vegetation → + snow depth → + winter soil temperature → + winter soil respiration → + C release → - NEE<br>• - Rodents → overall + ground temperature and + permafrost thaw → - soil respiration → + C release → - NEE<br>• - Rodents → overall + permafrost thaw → + lake/pond drainage (mostly in highlands) → + soil respiration → + CO^2 release → - NEE (but – CH_4 released)<br>• - Rodents → + field layer vegetation and + permafrost thaw → + lateral TOC transport → + C release → - NEE<br>• - Rodents → + field layer vegetation and + permafrost thaw → + lateral TOC transport → + brownification → - aquatic plant productivity → - C uptake → - NEE<br>• - Rodents → + field layer vegetation → changes in soil moisture → changes in soil respiration → changes in NEE	• + Insects → + BVOC → + SOA → + cloud cover → + diffuse radiation → + LUE → + plant productivity → + C uptake → + NEE<br>• + Insects → overall - air temperature → - permafrost thaw → - C release → + NEE<br>• + Insects → - vegetation growth → - vegetation cover and density → – snow interception → - ground temperature → - permafrost thaw and – soil respiration → - C release → + NEE<br>• + Insects → - vegetation growth → + snow masking → + snow season duration → - air and ground temperature → - permafrost thaw and – soil respiration<br>• + Insects → + grazing and browsing → overall - plant biomass and overall - permafrost thaw → - lateral TOC transport → - C release → + NEE<br>• + Insects → - vegetation growth → - C uptake → - NEE<br>• + Insects → - LAI → + ground warming and + permafrost thaw → + soil respiration → - NEE<br>• + Insects → + C released as BVOC → - NEE<br>• + Insects → overall - air temperature → - plant productivity → - C uptake → - NEE

	• + Plant productivity → + vegetation cover and density → + C emitted as BVOC → - NEE<br><br>• + Plant productivity → + plant biomass, + permafrost thaw and potentially + wetland areas → + lateral TOC transport → + C release → - NEE<br><br>• + Plant productivity → + lateral TOC transport → + brownification → - aquatic plant productivity → - C uptake → - NEE<br><br>• + Plant productivity → change in species composition → change in C sequestration rates<br><br>• + Plant productivity → + vegetation cover and density → changes in soil moisture → changes in soil respiration → changes in NEE				

BVOC	Biogenic Volatile Organic Compound
C	Carbon
CH_4	Methane
CO_2	Carbon dioxide
ET	Evapotranspiration
LAI	Leaf area index
LUE	Light use efficiency
NEE	Net primary production
TOC	Total organic carbon
SOA	Secondary organic aerosol

List of abbreviations used in Table S33.

Table S34. *Extreme biotic events as drivers of ecosystem change*

Ecosystem Component	**EXTREME BIOTIC EVENTS**	
	**Increasing insect outbreaks**	**Decreasing peaks in rodent population**
**Carbon Cycle**	• + Insect outbreaks → - vegetation cover and density → + albedo and – snow depth → - permafrost thaw → - soil respiration → - C release → + NEE<br>• + Insect outbreaks → overall – permafrost thaw → - lateral TOC transport → - C release → + NEE<br>• + Insect outbreaks → - vegetation cover and density → - snow depth → - winter ground temperature → - winter soil respiration → + NEE<br>• + Insect outbreaks → - vegetation cover and density → overall – ground temperature → - soil respiration → + NEE<br>• + Insect outbreaks → - vegetation cover and density overall - permafrost thaw → - C release → + NEE<br>• + Insect outbreaks → - plant biomass → - lateral TOC transport → - C release → + NEE<br>• + Insect outbreaks → - vegetation cover and density → - plant productivity → - C uptake → - NEE<br>• + Insect outbreaks → - vegetation cover and density → + albedo → - air temperature → - plant productivity → - C uptake → - NEE<br>• + Insect outbreaks → - vegetation cover and density → - LAI → + summer summer topsoil temperature → + summer topsoil respiration → - NEE<br>• + Insect outbreaks → - vegetation cover and density → changes in soil moisture → changes in soil respiration → changes in NEE	• - Rodent peaks → + field-layer vegetation → + C uptake → + NEE<br>• - Rodent peaks → + field-layer vegetation → + LAI → - summer topsoil temperature → - topsoil respiration → + NEE<br>• - Rodent peaks → overall + permafrost thaw → + subsidence (mostly in lowlands) → + soil moisture → + wetland vegetation and – dry hummock vegetation → + net C uptake → + NEE (but + CH_4 released)<br>• - Rodent peaks → - RD during rain events → - lateral TOC transport → - C release → + NEE<br>• - Rodent peaks → + field layer vegetation → - albedo → + air temperature → + plant productivity → + C uptake → + NEE<br>• - Rodent peaks → overall + permafrost thaw → + nutrient availability → + plant productivity → + NEE<br>• - Rodent peaks → overall + permafrost thaw → - soil respiration → + C release → - NEE<br>• - Rodent peaks → overall + permafrost thaw → + lake/pond drainage (mostly in highlands) → + soil respiration → + CO^2 release → - NEE (but – CH_4 released)<br>• - Rodent peaks → overall + permafrost thaw → + drainage (mostly in highlands) → - soil moisture → - plant productivity → - NEE<br>• - Rodent peaks → + field layer vegetation → + snow depth → + winter soil respiration → - NEE<br>• - Rodent peaks → + plant biomass and overall + permafrost thaw → + lateral TOC transport → + C release → - NEE<br>• - Rodent peaks → + plant biomass and overall + permafrost thaw → + lateral TOC transport → + brownification → - aquatic plant productivity → - C uptake → - NEE<br>• - Rodent peaks → + vegetation cover and density → changes in soil moisture → changes in soil respiration → changes in NEE

C	Carbon
CH_4	Methane
CO_2	Carbon dioxide
LAI	Leaf area index
NEE	Net primary production
TOC	Total organic carbon

List of abbreviations used in Table S34.

Table S35. *Local human activities as drivers of ecosystem change*

Ecosystem Component	**Local human Activities**			
	**Decreasing Herding**	**Decreasing black carbon deposition**	**Expanding infrastructures**	**Decreasing Fire wood out-take**
**Carbon Cycle**	• - Herding → - grazinf and browsing → + vegetation cover and density → + C uptake → + NEE<br>• - Herding → + vegetation cover and density → overall + air temperature → + plant productivity → + C uptake → + NEE<br>• - Herding → + vegetation cover and density → + LAI → - summer topsoil temperature → - topsoil respiration → + NEE<br>• - Herding → overall + permafrost thaw → + subsidence (mostly in lowlands) → + soil moisture → + wetland vegetation and - dry hummock vegetation → + net C uptake → + NEE (but + CH_4 released)<br>• - Herding → + vegetation cover and density → + snow depth → + ground temperature → + winter soil respiration → - NEE<br>• - Herding → overall + permafrost thaw → + lake/pond drainage (mostly in highlands) → + soil respiration → + CO^2 release → - NEE (but – CH_4 released)<br>• - Herding → overall + permafrost thaw → + drainage (mostly in highlands) → - soil moisture → - plant productivity → - NEE<br>• - Herding → + vegetation cover and density → overall + permafrost thaw → + soil respiration → + C release → - NEE<br>• - Herding → + plant biomass and + permafrost thaw → + lateral TOC transport → + C release → - NEE<br>• - Herding → + plant biomass and + permafrost thaw → + lateral TOC transport → + brownification → - aquatic plant productivity → - C uptake → - NEE	• - BC → + soil moisture during the growing season → + plant productivity → + C uptake → + NEE<br>• - BC → overall - plant productivity → - plant biomass → - lateral TOC transport → - C release → + NEE<br>• - BC → + lake-ice duration → - microbial activity → - CO^2 and CH_4 release → + NEE<br>• - BC → + albedo and + snow cover duration → - air temperature → - plant productivity → - C uptake → - NEE<br>• - BC → + lake-ice duration → - aquatic plant productivity → - C uptake → - NEE<br>• - BC → + soil moisture, RD and GWF in late spring and summer → + lateral TOC transport → + C release → - NEE<br>• - BC → + soil moisture, RD and GWF in late spring and summer → + lateral TOC transport → + brownification → - aquatic plant productivity → - C uptake → - NEE	• + Infrastructures → + grazing pressure in certain areas → - vegetation cover and density → - C uptake → - NEE<br>• + Infrastructures → - vegetation cover and density → - C uptake → - NEE<br>• + Infrastructures → + permafrost thaw → + C release → - NEE<br>• + Infrastructures → changes in soil moisture → changes in soil respiration → changes in NEE<br>• + Infrastructures → changes in soil moisture → changes in plant species composition → changes in C uptake rates → changes in NEE	• - Fire wood out-take → + forest cover and density → + C uptake → + NEE<br>• - Fire wood out-take → overall + permafrost thaw → + soil respiration → + C release → - NEE<br>• - Fire wood out-take → overall + permafrost thaw → + lake/pond drainage (mostly in highlands) → + soil respiration → + CO^2 release → - NEE (but – CH_4 released)<br>• - Fire wood out-take → + plant biomass and overall + permafrost thaw → + lateral TOC transport → + C release → - NEE<br>• - Fire wood out-take → + plant biomass and overall + permafrost thaw → + lateral TOC transport → + brownification → - aquatic plant productivity → - NEE

	• - Herding → + vegetation cover and density → changes in soil moisture → changes in soil respiration → changes in NEE			

BC	Black carbon
C	Carbon
CH_4	Methane
CO_2	Carbon dioxide
LAI	Leaf area index
GWF	Ground water flow
NEE	Net primary production
RD	River discharge
TOC	Total organic carbon

List of abbreviations used in Table S35.

A specific example

Example: Future changes in the arctic marine ecosystems.

The Arctic marine ecosystems are undergoing major changes and these changes are very likely to continue over the coming decades and centuries. We know that there are several drivers dictating these changes through multiple and complex processes. Mainly due to a lack of studies, model limitations and time constraints, we do not know quantitatively the magnitude of the impact that each driver and each process will exert on each ecosystem component of the arctic marine environments. However, there are scientists who have extensively studied these drivers and components, and thus can have a better sense of the magnitude of these impacts – the participants in this Expert Assessment.

The experts cannot accurately quantify these impacts in absolute terms, but they might be able to give an indication of their importance relative to one another.
Questions 1A and ***1B*** are intended to collect the information needed to rank/order the different drivers according to their potential impact on the ecosystem component concerned for the periods 2020 – 2040 and 2040 – 2100, respectively:

Q1.1A. Rank the likely impact of decreasing sea-ice extent on the primary productivity in the Arctic Ocean for the period 2020 - 2040. *

	1	2	3	4	5	6	7	8	9	
Low impact	○	○	○	○	○	○	○	○	○	High impact

Specify what processes contribute the most to the impact.

Your answer

Q1.1B. Rank the likely impact of decreasing sea-ice extent on the primary productivity in the Arctic Ocean for the period 2040 - 2100. *

	1	2	3	4	5	6	7	8	9	
Low impact	○	○	○	○	○	○	○	○	○	High impact

Specify what processes contribute the most to the impact.

Your answer

Naturally, the impacts of decreasing sea-ice extent (driver of change) on the primary production (ecosystem component) in the Arctic Ocean will likely vary largely among species, geographical areas, temporal scales etc. Nevertheless, it is reasonable to assume that a decrease in sea-ice extent will likely have a general impact on the primary production of the Arctic Ocean in the coming century. We want to emphasize that this *first question* is intended to identify the main drivers of change and for this purpose **generalizations are needed**.
Another important consideration is that each driver can have direct and indirect effects on an ecosystem component: a decrease in sea ice extent directly decreases primary production by decreasing the habitat of some sea-ice algae species, but it increases primary production due to the larger amount of light reaching the ocean's surface. When completing the survey, **you must consider both the direct and indirect processes** arising from each changing driver. Finally, when ranking the impact of a given driver

on an ecosystem component, you should assume as a baseline that conditions of any other drivers remain unchanged.

Thus, in order to answer the question the expert must decide whether the driver *decreasing sea-ice extent,* in this case*,* will have any impacts on the primary production of the Arctic Ocean for the periods indicated in the question:

- if the expert thinks that this driver will have a neglectable impact, the answer will be 1.
- if the expert thinks that this driver will have the larger impact, the answer will be 9.
- else, the expert has to estimate the potential impact in comparison with the other drivers.

The expert must bare in mind that if the impacts of driver *A* are thought to be more important than those of driver *B,* driver A **must always** be given a higher rank than driver *B*. Thus, we recommend to use the *Tables* provided above in order to get an overview of the different drivers and processes involved prior to completing the survey. Nevertheless, you will always be able to come back to modify your answers. Also notice that **the importance of a specific driver can change from one period to another** because of the achievement of a new equilibrium, and the emergence of new drivers of change, for example.

The experts are also encouraged to specify what processes they think will exert the greatest effect on the component concerned. This can be done in the sub-question following Questions 1A and 1B, and a possible response style could be:

"I believe that the major processes contributing to the impact would be: increase in phytoplankton biomass due to the light intensity and ocean mixing, and to a lesser extent the reduction in ice-algae. The overall result, however, would be an increase in primary production."

In ***Question 2,*** the experts are asked to indicate, from 1 to 9, how well studied are the potential impacts of a driver on the ecosystem component concerned. Thus, the experts have to rank-order drivers which they 'would like to further investigate' in order to improve future predictions of ecosystem change in the study area.

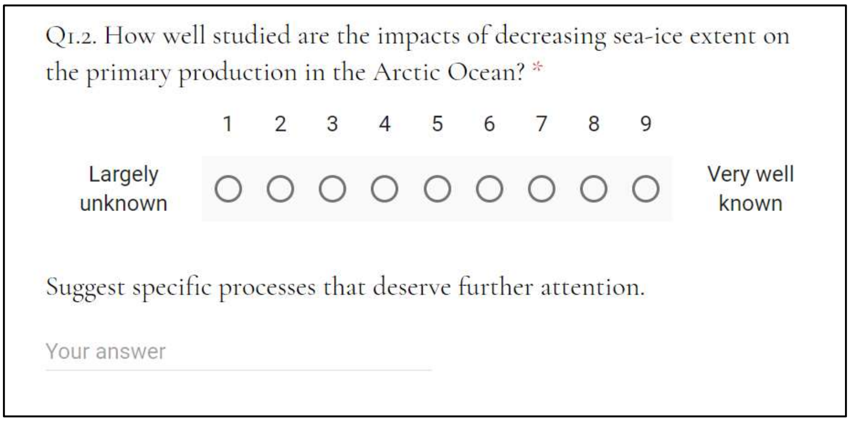
Q1.2. How well studied are the impacts of decreasing sea-ice extent on the primary production in the Arctic Ocean? *

	1	2	3	4	5	6	7	8	9	
Largely unknown	○	○	○	○	○	○	○	○	○	Very well known

Suggest specific processes that deserve further attention.

Your answer

The decrease in sea-ice extent has been extensively studied and reasonably well modelled in the recent years and therefore the experts will likely decide not to consider this driver and the processes involved as poorly studied (1 or close). However, sea-ice dynamics in itself still involve relatively important uncertainties which might influence the magnitude and timing of a suite of processes and thus the final response of marine autotrophs in the future. Moreover, some of the multiple direct and indirect processes triggered by the decrease in sea-ice and their consequences for the primary production in the Arctic Ocean might be poorly constrained. Hence, the experts will likely decide not to consider this driver and the processes involved as very well studied (9 or close). Instead, the experts might chose a number between 3 and 7, and they can specify what specific processes they think deserve further attention in the sub-question following Question 1.2. A suitable answer could be:

"*I think that more research should be conducted on constraining the effect of the sea-ice-induced increase in cloud cover on light intensity and thus primary production, as it could play an important*

role in the future phytoplankton dynamics in the Arctic Ocean. Moreover, the future decrease rate in sea-ice should be better studied as it seems to be decreasing faster than most models projected".

In **Question 3**, you are asked to rank your expertise in the issues addressed in ***Questions 1*** and ***2.***

Q1.3. Rank your expertise in Q1.1 and Q1.2: *

	Not familiar	Little knowledge	Some knowledge	Good knowledge	Very good knowledge
Q1.1 - Q1.2					

An expert on an ecosystem component (primary production, in this example) might not have been directly working with sea-ice dynamics in depth. However, the knowledge on primary production dynamics might still allow you to know what effects a change in a given driver can have on the component you have expertise in. In this question, we want to know 'how sure the expert is that their answers on the previous questions are certain'.

At the end of the survey, there are two final questions where the expert can suggest relevant drivers not included in the survey and provide with additional thoughts on the survey: general thoughts, reasoning behind unanswered questions, or additional information on answered questions.

Appendix S2. Data analysis.

All responses belonging to the same group of experts were gathered and analyzed together using the same methodology. Before the analysis, we identified twelve cases in which the response to a specific question appeared highly anomalous. We provided the corresponding experts with a new detailed explanation of the question and asked them to confirm that they meant what they responded. In all cases, they responded that they had not correctly understood the question, and provided revised answers.

The experts were explicitly asked to rank the importance of each driver relative to each other driver. This assumes that the most and least important drivers should be assigned the highest (9) and lowest (1) possible ranks, respectively. However, we received several responses that were not given accordingly, and did not use the entire scale range in their responses. In order to correct for this and make responses among experts comparable, we normalized them on a 0-10 scale using equation 1 below:

$$x' = (x_i - xmin_i)/(xmax_i - xmin_i)*10 \qquad \text{Equation 1}$$

where x' is the normalized value, x_i is the value being normalized, $xmin_i$ and $xmax_i$ are the minimum and maximum scores assigned by the expert i throughout the entire survey when evaluating the variable importance (Questions 1a and 1b), and 10 is for magnitude conversion.

The scores for Question 2 were inverted in order to convert awareness into novelty, which is indicative of how new, or understudied, are the ecosystem impacts of a given driver. Subsequently, the scores were normalized on a 0-10 scale in order to make them comparable to the variable importance. In this case, the maximum and minimum possible ranks were not necessarily representative of the best and least studied drivers, and so the following equation was used:

$$x' = (x - xmin)/(xmax-xmin)*10 \qquad \text{Equation 2}$$

where x' is the normalized value, x is the value being normalized, xmin and xmax are the minimum (9) and maximum (1) possible scores, and 10 is for magnitude conversion.

All responses for each variable were aggregated by averaging the normalized scores. It is important to emphasize that the importance scores reported in the text refer to relative importance, meaning that a certain change in the absolute impact of a given driver can be translated into a relative increase or decrease depending on magnitude and direction of the changes perceived in the impacts exerted by the rest of the drivers. In reporting results, responses with self-rated expertise of 1 (not familiar) were excluded. This addressed imperfections in the expert selection process (Schuur et al. 2013), and increased the quality of the results by ensuring that all estimates were provided by experts with expertise in each specific driver.

Appendix S3. Overview of the groups of experts, and the experts' estimates of importance and novelty for the top 10 most important drivers for each ecosystem component.

Table S36. Overview of the groups of experts participating in the Expert Assessment.

Ecosystem component	n respondents	Country respondents	Gender respondents	Self-reported expertise for the top 10 most important drivers (1-5)	n responses excluded due to expertise <2 (%)*
Local Climate	4	Poland,<br>Sweden (n=2)<br>United Kingdom	Female (n=2)<br>Male (n=2)	2.9	3 (3.8)
Permafrost	6	Sweden (n=6)	Female (n=2)<br>Male (n=4)	3.2	17 (14.1)
Hydrology	5	Canada<br>Sweden (n=3)<br>United States	Female (n=1)<br>Male (n=4)	3.6	0
Vegetation	6	Denmark<br>Norway<br>Sweden (n=2)<br>United Kingdom[a] (n=2)<br>Russia[a]	Female (n=1)<br>Male (n=5)	3.3	8 (6.6)
Carbon Cycle	6	Sweden (n=3)<br>Denmark (n=2),<br>United Kingdom	Female (n=4)<br>Male (n=2)	3.4	12 (10)

* Responses with a self-reported expertise of 1 (not familiar) were excluded from the results reported in the text.

[a] One respondent belongs to Russian and English universities.

Table S37. Local climate experts' estimates.

Period 2020-2040			Period 2040-2100		
**Driver**	**Mean ± SD importance**	**Mean + SD novelty**	**Driver**	**Mean ± SD importance**	**Mean + SD novelty**
Air Temperature	8.8 ± 2.5	4.1 ± 1.7	Air Temperature	9.5 ± 1	4.1 ± 1.7
Rainfall	7.6 ± 1.7	5.3 ± 1	Winter Warming Events	7.8 ± 1.8	5.3 ± 1.3
Cloud Cover	7.2 ± 3.5	6.7 ± 1.2	Rainfall	7.2 ± 2.2	5.3 ± 1
Snow Cover	7 ± 3.1	6.6 ± 1.3	Lake-Ice Duration	6.7 ± 2.6	5.9 ± 1
Winter Warming Events	6.7 ± 3.1	5.3 ± 1.3	Snow Cover	6.5 ± 1.9	6.6 ± 1.3
Evapotranspiration	6.3 ± 4.4	5.9 ± 1	Evapotranspiration	6.3 ± 2.9	5.9 ± 1
Lake-Ice Duration	6.1 ± 3	5.9 ± 1	Droughts	6 ± 2.3	7.8 ± 1
Snow Depth	5.7 ± 3.3	6.9 ± 1.3	Snow-Water Equivalent	4.8 ± 2.6	7.2 ± 1.3
Droughts	5.5 ± 2.9	7.8 ± 1	Black Carbon	4.8 ± 3.9	5 ± 1.4
Soil Moisture	4.6 ± 4.5	6.9 ± 1.3	Soil Moisture	4.6 ± 2.5	6.9 ± 1.3
Top 10 mean	*6.6 ± 3.2*	*6.1 ± 1.2*	*Top 10 mean*	*6.4 ± 2.4*	*6 ± 1.2*

Table S38. Permafrost experts' estimates.

Period 2020-2040			Period 2040-2100		
**Driver**	**Mean ± SD importance**	**Mean + SD novelty**	**Driver**	**Mean ± SD importance**	**Mean + SD novelty**
Air Temperature	10 ± 0	3.8 ± 1.8	Air Temperature	9.3 ± 1.6	3.8 ± 1.8
Snow Cover	9 ± 1.3	4.2 ± 1.4	Snow Cover	8.9 ± 0.9	4.2 ± 1.4
Snow Depth	8.6 ± 2	3.5 ± 2.1	Snow Depth	8.6 ± 2	3.5 ± 2.1
River Discharge - Groundwater Flow	8.5 ± 1	5.9 ± 2.2	Rainfall	8.5 ± 1.2	5.3 ± 1.3
Winter Warm Events	8.1 ± 2.2	5.8 ± 2.1	Snow-Water Equivalent	8.4 ± 2.4	6.9 ± 1.7
Soil Moisture	8.1 ± 2.1	6.3 ± 1.8	River Discharge - Groundwater Flow	8.1 ± 1.4	5.9 ± 2.2
Snow-Water Equivalent	8 ± 2.1	6.9 ± 1.7	Winter Warming Events	7.7 ± 1.9	5.8 ± 2.1
Rainfall	7.6 ± 1.3	5.3 ± 1.3	Soil Moisture	7.3 ± 2.4	6.3 ± 1.8
Plant Productivity	6.4 ± 1.5	5 ± 1.4	Evapotranspiration	6.7 ± 2.9	7.9 ± 1.5
Droughts	6.3 ± 2.5	7 ± 0.9	Droughts	6.3 ± 2.5	7 ± 0.9
Top 10 mean	*8.1 ± 1.6*	*5.4 ± 1.7*	*Top 10 mean*	*8 ± 1.9*	*5.7 ± 1.7*

Table S39. Hydrology experts' estimates.

Period 2020-2040			Period 2040-2100		
**Driver**	**Mean ± SD importance**	**Mean + SD novelty**	**Driver**	**Mean ± SD importance**	**Mean + SD novelty**
Rainfall	9.5 ± 0.7	5.8 ± 1.8	Rainfall	9.8 ± 0.6	5.8 ± 1.8
Snow Cover	8.4 ± 1.8	5.3 ± 1.5	Snow Cover	8.9 ± 1.9	5.3 ± 1.5
Snow Depth	7.8 ± 1.6	5.5 ± 1.5	Winter Warming Events	8 ± 3.1	7.8 ± 0.8
Evapotranspiration	7.8 ± 2.8	6.5 ± 1.3	Snow Depth	7.7 ± 1.6	5.5 ± 1.5
Snow-Water Equivalent	7.8 ± 3	5.8 ± 1.5	Evapotranspiration	7.5 ± 2.7	6.5 ± 1.3
Lake-Ice Duration	7.7 ± 2	6 ± 1.3	Snow-Water Equivalent	7.5 ± 3	5.8 ± 1.5
Air Temperature	7.4 ± 3.6	6 ± 2.2	Air Temperature	7.5 ± 3.6	6 ± 2.2
Extreme Rainfall Events	7.3 ± 3	8 ± 0.9	Droughts	7.5 ± 3.5	7.5 ± 1.7
Droughts	7.2 ± 3.5	7.5 ± 1.7	Extreme Rainfall Events	7.2 ± 3.2	8 ± 0.9
Winter Warming Events	7.1 ± 3	7.8 ± 0.8	Plant Productivity	7.1 ± 2.9	7.5 ± 1.2
Soil Moisture	7.1 ± 1.9	6.5 ± 1.5	Lake-Ice Duration	7 ± 3	6 ± 1.3
Top 10 mean	7.7 ± 2.4	6.4 ± 1.5	Top 10 mean	7.8 ± 2.5	6.5 ± 1.4

Table S40. Vegetation experts' estimates.

Period 2020-2040			Period 2040-2100		
**Driver**	**Mean ± SD importance**	**Mean + SD novelty**	**Driver**	**Mean ± SD importance**	**Mean + SD novelty**
Air Temperature	8.4 ± 1.6	2.1 ± 1.5	Air Temperature	8.7 ± 2.1	2.1 ± 1.5
Snow Cover	7.9 ± 1.7	4.8 ± 1	Snow Cover	7.9 ± 1.1	4.8 ± 1
Herding	7.8 ± 3	3.5 ± 2.2	Winter Warming Events	7.9 ± 4	5.2 ± 2.2
Winter Warming Events	7.5 ± 3.9	5.2 ± 2.2	Herding	7.8 ± 2.2	3.5 ± 2.2
Insect Population	6.8 ± 2.4	4.4 ± 2	Rainfall	7.2 ± 1.8	4.6 ± 2.1
Rodents Population	6.4 ± 2.3	2.3 ± 1.2	Insect Population	6.5 ± 2.8	4.4 ± 2
River Discharge - Groundwater Flow	6.4 ± 2.5	5.6 ± 1.7	Evapotranspiration	6.5 ± 2.9	6.8 ± 1.3
Evapotranspiration	6.3 ± 2.6	6.8 ± 1.3	Soil Moisture	6.2 ± 2.9	7.2 ± 1
Snow Depth	6 ± 1.8	5 ± 2.1	Snow Depth	6.1 ± 2.4	5 ± 2.1
Rainfall	5.8 ± 1.6	4.6 ± 2.1	Rodents Population	5.9 ± 1.7	2.3 ± 1.2
Top 10 mean	*6.9 ± 2.3*	*4.4 ± 1.7*	*Top 10 mean*	*7 ± 2.4*	*4,6 ± 1.7*

Table S41. Carbon cycle experts' estimates.

Period 2020-2040			Period 2040-2100		
**Driver**	**Mean ± SD importance**	**Mean + SD novelty**	**Driver**	**Mean ± SD importance**	**Mean + SD novelty**
Lake Ice Duration	8.2 ± 1.6	8.3 ± 0.6	Air Temperature	9.4 ± 0.9	3.5 ± 1.5
Air Temperature	8.1 ± 1.9	3.5 ± 1.5	Plant Productivity	8.9 ± 1.4	4.4 ± 2.1
Plant Productivity	7.7 ± 2.1	4.4 ± 2.1	Snow Cover	8.6 ± 1.6	5.8 ± 1.8
Snow Cover	7 ± 2	5.8 ± 1.8	Lake Ice Duration	8.4 ± 1.5	8.3 ± 0.6
Soil Moisture	6.8 ± 2.7	5.4 ± 1.5	Winter Warming Events	7.7 ± 2.6	6 ± 2.5
Winter Warming Events	6.8 ± 3	6 ± 2.5	Insect Outbreaks	7.5 ± 2.7	6.3 ± 1.1
Dissolved Organic Carbon	6.5 ± 1.8	4.7 ± 2.6	Rainfall	7.5 ± 1.5	6.7 ± 2
Active Layer Thickness	6.3 ± 2.1	4.4 ± 1.4	Soil Moisture	7.3 ± 3	5.4 ± 1.5
Insect Outbreaks	5.9 ± 3	6.3 ± 1.1	Dissolved Organic Carbon	7.2 ± 1.1	4.7 ± 2.6
Evapotranspiration	5.9 ± 2.4	6 ± 2.3	Evapotranspiration	7.2 ± 1.2	6 ± 2.3
Top 10 mean	*6.9 ± 2.26*	*5.5 ± 1.7*	*Top 10 mean*	*8 ± 1.8*	*5.7 ± 1.8*

Appendix S4. Important future studies suggested by the different groups of experts.

Table S42. Important future studies suggested by local climate experts involving the different local climate research priorities (shaded) and other important drivers of local climate change in the Torneträsk area.

Most important drivers and Research Priorities	Suggested studies
Rainfall	• The effects of rain on snow events modifying the snow cover conditions and thus the winter climate.<br>• The spatial and temporal effects of the rainfall-induced increases in evapotranspiration and vegetation on the surface energy balance (latent heat and albedo effects).
Cloud cover	• The positive and negative effects of changes in cloud cover and type on surface air temperature.
Snow cover	/
Winter warming events	• The relation between micrometeorology (e.g. lapse rates, boundary layer stability), atmospheric circulation/air masses and climate change.
Evapotranspiration	• The inclusion of evapotranspiration-climate interactions in models.
Lake ice duration	• The future changes in lake-ice duration and its effects on the local climate of the Torneträsk area.
Droughts	• Future changes in frequency of Scandinavian (high-pressure) blocking of jet stream and their relation with local meteorology
Air temperature	• Feedbacks between air temperature and snow duration, lake-ice duration and vegetation.
Snow depth	/
Soil moisture	• Soil moisture-induced changes in BVOC emissions and its effects on local climate.<br>• Soil moisture induced changes in evapotranspiration and atmospheric water vapour content and its effects on local climate.
Snow-water equivalent	• The spatial distribution of SWE and its impact on local climate via evapotranspiration and albedo (snow-cover duration and vegetation dynamics).
Black carbon	• The effects of changes in black carbon deposition and albedo of snow.

Table S43. Important future studies suggested by permafrost experts involving the different permafrost research priorities (shaded) and other important drivers of permafrost change in the Torneträsk area.

Most important drivers and Research Priorities	Suggested studies
RD-GWF	• Site specific studies about GWFs effect on permafrost.<br>• Development of a river discharge monitoring program in several of the rivers in the study area.<br>• The impacts of extreme discharge events (both through intense snow melting and during periods of intense precipitation) on lowland permafrost.
Winter warming events	• The potential positive (decrease in snow cover duration, increase in winter air temperature, etc) and negative (e.g. reduction in the insulating capacity of the snow pack) feedbacks of winter warming events to permafrost temperatures in the area.
Soil moisture	• The spatial and temporal changes on soil moisture conditions and its impacts on ground thermal conductivity and permafrost temperatures.<br>• Development of a monitoring program of the mountain based permafrost.
Snow-water equivalent	• Effects on future snow-water equivalent on the insulation capacity of the snow pack.
Rainfall	• Studies on the effects of seasonal changes in precipitation and extreme events on permafrost dynamics.<br>• Detailed analyses of precipitation patterns during all the historical record period and within a specific new project.<br>• The effects of future precipitation changes on mountain permafrost.
Plant productivity	• Impacts of future vegetation-snow interactions in the alpine-subalpine permafrost.
Droughts	/
Evapotranspiration	• Studies on the balance and timing between increased precipitation and evapotranspiration and impacts on the soil and ground's hydrological regime and thermal conductivity.<br>• Interactions between increased evapotranspiration and surface air temperature, and their impacts on permafrost.
Air temperature	• Research about seasonality/variations, as well as extreme events.<br>• Studies on soil and ground water temperature.<br>• Studies on soil water content and temperature in the active layer.<br>• More studies need to be conducted in mountain permafrost areas.

Snow cover	• Balance between increasing air temperatures and snow precipitation and consequences for snow cover duration and albedo.
Snow depth	• New studies of snow depths and time with cover at strategic places within the catchment. Link these to the historical data from the weather station.<br>• Balance between increasing snow precipitation, the shortening of the snow season and the increase of winter warming events.

Table S44. Important future studies suggested by hydrology experts involving the different hydrology research in the Torneträsk area.

Research Priorities	Suggested studies
Rainfall	• Need for a decrease in the uncertainties on the timing and magnitude of future precipitation changes.<br>• Integration of the different direct and indirect effects of rainfall on the hydrologic conditions of the area.<br>• The interaction between altered discharge and the temperature responses (evapotranspiration, length of ice free season) and how that affect water transit times in lakes and rivers.
Snow cover	• Soil moisture and summer wetland conditions due to changes in snow melt magnitude.<br>• Balance between shorter snow-pack periods and anticipated greater snowfall - the net effect on peak snowpack depth and the timing of snowmelt.<br>• The balance between warming air temperatures and light conditions, and its effects on snowmelt and flooding.<br>• The spatial variability in snow cover distribution and its impacts on hydrological processes.<br>• Implications of reduced snow cover duration on stream ecological processes.
Snow depth	• Wetland processes related to altered snow depth.<br>• The balance between shorter snow period and greater winter snow precipitation and its influence on the magnitude, timing and rate of snowmelt.
Evapotranspiration	• Impacts on stream ecological processes.<br>• Regional-scale studies on the effects of increasing evapotranspiration on the hydrological regime of the study area.<br>• The effects of increasing evapotranspiration and river discharge and the impacts on the connectivity between the terrestrial and aquatic environments.

Snow-water equivalent	• Similar studies as mentioned for changes in snow depth.
Lake ice duration	• Impact of changes in lake-ice duration on different systems (morphometry and altitude) and interactions with changes in runoff.<br>• Impacts of decreasing lake-ice duration on light penetration and water temperatures (thus aquatic productivity), and evapotranspiration.
Air temperature	• Need for more studies at regional scales.
Extreme rainfall events	• The interaction between altered discharge and length of ice free season and how that affect water transit times in lakes and rivers.<br>• Impacts on the functioning of the streams and their chemistry.<br>• Impacts on stream ecology and other processes such as greenhouse gas losses, element losses and physical impacts on the stream channels.
Droughts	• Long-term studies on stream ecology and biogeochemistry to better understand the ecosystem response to this climatic events.<br>• Hydrologic connectivity to biotopes.
Winter warming events	• High resolution measurements of stream flow during winter time.
Plant productivity	• Studies on the long-term consequences for aquatic ecosystems, mainly on biological processes as well as element fluxes.<br>• Impacts of evapotranspiration and interception effects on GWF RD.
Soil moisture	/

Table S45. Important future studies suggested by vegetation experts involving the different vegetation research priorities (shaded) and other important drivers of vegetation change in the Torneträsk area.

Most important drivers and Research Priorities	Suggested studies
Winter warming events	• Simulations of increased frequency and magnitude of events.<br>• Need for greater observational power from local people together with better weather forecasting so scientists can be forewarned to be present to observe an event.<br>• Impacts on spring phenology in this sub-Arctic region.<br>• Impacts on vegetation other than dwarf shrub heathland.

	• Capacity of mortality by winter warming events to open space for species invasion.
RD-GWF	• Need for better precipitation modelling and surface filtration under various moisture and vegetation regimes.
Evapotranspiration	• Estimating the negative feedback (at km and cm square scales) via ET for different vegetation types, communities and species.<br>• Effects of evapotranspiration on drought and the resulting reduction in plant productivity.
Snow depth	• Studies of decreased soil moisture on terrestrial upland ecosystems.<br>• The spatial distribution of snow depth and the layers within it.<br>• Impacts on nutrient cycling and availability of nutrients to plants in spring.
Soil moisture	• Need for more accurate projections of future precipitation, particularly for the longer term.<br>• Impacts of soil moisture on primary productivity.<br>• Impacts of soil moisture changes in plant species with different rooting zones.<br>• Studies with experimental changes in soil moisture and impact on vegetation in the area.
Air temperature	• Studies of the migration of species and the adaptation of the existing ones.<br>• Interactions between heat stress, insect outbreak and moisture stress<br>• Studies on the land surface albedo and plant evapotranspiration feedback effect.<br>• Impact of invasive / more southerly distributed species.<br>• Interactions between increasing air temperature and elevated CO_2.<br>• Studies on the acclimatization to long-term warming vs shorter term warming.
Snow cover	• The balance between increased (and earlier) growing season and moisture stress.<br>• Impacts of snow cover changes on growing season length and hence productivity.
Herding	• Studies using model scenarios of different future managements and targeting different vegetation types.<br>• Grazing impacts on biodiversity.
Insect population	• There is a need for more information from shrubs and herbaceous vegetation.<br>• Modelling studies of future insect outbreaks.<br>• Studies of combined herbivore effects (insects, rodents, ungulates)

Rodents population	• Combined studies including multiple herbivore species
Rainfall	• The magnitude and distribution of future precipitation changes and its impacts on different vegetation types.<br>• The multiple interactions of vegetation with moisture stress, insect damage and fire.<br>• The response of the more productive invasive species to changes in rainfall.<br>• The effects of rainfall on terrestrial upland ecosystems.

Table S46. Important future studies suggested by carbon cycle experts involving the different carbon cycle research priorities (shaded) and other important drivers of carbon cycle change in the Torneträsk area.

Most important drivers and Research Priorities	Suggested studies
Lake-ice duration	• In small lakes, studies should focus on the key processes within the water-body itself (e.g. thermal stratification, the timing and seasonal patterns of primary productivity etc). In larger lakes (e.g. Torneträsk) these should focus on the implications for the adjacent shoreline and terrestrial environments (e.g. erosion; thermal regime), and possibly for deltaic (Abisko Jokk) and sedimentary processes.<br>• Effects on stratification and water circulation patterns, and their implications for C cycling that could be profound in a water body the size of Torneträsk.
Snow cover	• Implications, at landscape scale, for plant phenology and herbivores, and for hydrology.<br>• Long-term continuous measurements of CO_2, CH_4 and BVOC (preferably eddy covariance) to understand the role of winter processes in the carbon cycle.<br>• Redistribution and modification of snow by wind, and the implications for grond temperatures and soil organic carbon decomposition in winter.
Soil moisture	• The implications for GWF, RD and downstream water chemistry.<br>• The implications, at landscape scale, of changes in soil moisture for plant and soil processes (some parts of the landscape will be very sensitive/vulnerable, while others will be more resilient).
Winter warming events	• Effects on surface energy budget.<br>• Effects of rain on snow events on vegetation dynamics.

Insect outbreaks	• The effects of insect outbreaks on nutrient recycling, as well as the extent to which mountain birch can survive increased frequencies and/or intensities of outbreaks.<br>• Long-term studies that assess C cycle processes during the recovery period.<br>• Studies on the BVOC emissions during outbreak and during the years following outbreak.
Evapotranspiration	• Understanding the potential for shifts in evapotranspiration to cause either (i) waterlogging or (ii) water deficits, in contrasting landscape positions and on contrasting timescales.<br>• Understanding the links between evapotranspiration and GWF and RD, which integrates both temperature and precipitation.<br>• Shifts in the delivery, and fate, of terrestrial C into GWF and RD.<br>• Alterations in soil thermal and moisture regimes and their effects on both plants and soil organisms.
Rainfall	• Studies on the seasonal shifts in rainfall (and rain-on-snow events).<br>• The shifts in the delivery and fate of terrestrial C into GWF and RD.<br>• Alterations in soil thermal and moisture regimes and their effects on both plants and soil organisms, as well as their effects on permafrost.
Air temperature	• The coupling between changes in air temperature, vegetation structure, and soil/permafrost thermal and hydrological regimes.
Plant productivity	• Plant-soil interactions, rhizosphere processes and changes in soil organic matter dynamics associated with increasing plant productivity.<br>• The linkages between plant and soil interactions in the terrestrial part of the catchment, and the implications for surface runoff timing, quantity and chemistry.
DOC concentrations	• The processes in soils and sediments that produce DOC, and the fate of DOC in lakes and rivers.
Active layer thickness	• The impacts of human disturbance on the spatially limited areas of lowland permafrost.
